# Creatine Promotes Endometriosis by Inducing Ferroptosis Resistance via Suppression of PrP

**DOI:** 10.1002/advs.202403517

**Published:** 2024-08-09

**Authors:** Siman Chen, Xiaoqian Ma, Yukai Liu, Zhiqi Zhong, Chunyan Wei, Mingqing Li, Xiaoyong Zhu

**Affiliations:** ^1^ Laboratory for Reproductive Immunology Hospital of Obstetrics and Gynecology Fudan University Shanghai 200090 P. R. China; ^2^ Fujian Provincial Key Laboratory of Reproductive Health Research Department of Obstetrics and Gynecology The First Affiliated Hospital of Xiamen University School of Medicine Xiamen University Fujian 361102 P. R. China; ^3^ Xinglin College Nantong University Nantong 226001 P. R. China; ^4^ Shanghai Key Laboratory of Female Reproductive Endocrine Related Diseases Fudan University Shanghai 200090 P. R. China

**Keywords:** creatine, endometriosis, ferroptosis, iron uptake, prion protein (PrP)

## Abstract

Endometriosis, a chronic inflammatory disease, significantly impairs the quality of life of women in their reproductive years; however, its pathogenesis remains poorly understood. The accumulation of retrograde menstruation and recurrent bleeding fosters a high‐iron environment in ectopic lesions, triggering ferroptosis in ectopic endometrial stromal cells (EESCs), thereby hindering the establishment of endometriosis. However, abnormal EESCs demonstrate resistance to ferroptosis in high‐iron environments, promoting the progression of this disease. Here, novel findings on the accumulation of creatine, derived from endogenous synthesis, in both peritoneal fluid and EESCs of patients with endometriosis are presented. Creatine supplementation reduces cellular iron concentrations, mitigating oxidative stress and lipid peroxidation, thereby enhancing cell viability and preventing ferroptosis under high‐iron conditions. Utilizing the drug affinity–responsive target stabilization (DARTS) assay, prion protein (PrP) as a potential creatine‐sensing protein is identified. Mechanistically, creatine binds to the active site of PrP, inhibits the conversion of trivalent iron to divalent iron, and decreases iron uptake, promoting the tolerance of EESCs to ferroptosis. This interaction contributes to the development of endometriosis. The novel association between creatine and ferroptosis provides valuable insights into the role of creatine in endometriosis progression and highlights its potential as a therapeutic target for endometriosis.

## Graphical abstract

Endometrial stromal cells (ESCs) within endometriotic lesions express glycine amidinotransferase (GATM) at high levels and accumulate creatine. Creatine binds to the intracellular sensing protein prion protein (PrP) and causes functional abnormalities, inhibiting the conversion of trivalent iron to divalent iron and iron intake. Consequently, creatine induces ferroptosis resistance in ESCs and triggers the onset and progression of endometriosis.

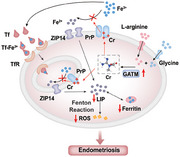

## 1 Introduction

Endometriosis (EMs), an estrogen‐dependent, chronic inflammatory disease characterized by the growth of endometrium‐like tissues outside the uterine cavity, affects up to 10% of women of reproductive age.^[^ ^1^ ^]^ The clinical symptoms of endometriosis, including progressive dysmenorrhea, chronic pelvic pain, deep dyspareunia, and infertility, significantly impact the quality of life.^[^ ^2^ ^]^ The etiology of EMs remains elusive, complicating its diagnosis and treatment.

Nevertheless, the theory of retrograde menstruation, first proposed by Sampson in 1921, which involves the reflux of menstrual debris into the peritoneal cavity through the fallopian tubes resulting in implantation, is widely accepted.^[^ ^3^ ^]^ This retrograde menstruation can substantially increase heme and iron levels in EMs lesions.^[^ ^4^ ^]^ We previously demonstrated that elevated heme impairs macrophage phagocytosis and induces progesterone resistance in ectopic endometrial stromal cells (EESCs), facilitating EMs development.^[^ ^5^ ^]^ Heme lysis is a source of iron overload, inducing ferroptosis of EESCs^[^ ^6^ ^]^–a type of iron‐mediated non‐apoptotic programmed cell death characterized by lipid hydroperoxide accumulation.^[^ ^7^ ^]^ Activation of ferroptosis reportedly results in the nonapoptotic destruction of certain cancer cells, whereas resistance to ferroptosis promotes tumor growth and metastasis.^[^ ^8^ ^]^ While traditionally considered benign disease, EMs exhibit several characteristics, including proliferation, infiltration, metastasis, and recurrence, similar to that of invasive cancers.^[^ ^9^ ^]^ EESCs from patients with endometriosis exhibit resistance to ferroptosis, enabling them to survive, implant, and proliferate within the peritoneal cavity.^[^ ^10^ ^]^ However, the molecular mechanisms underlying the survival of EESCs in patients with endometriosis under ferroptotic pressure remain unclear.

Creatine (Cr) (also known as α‐methyl guanidine acid), a naturally occurring nitrogen‐containing organic acid, is commonly found in diets rich in fish, poultry, and red meat,^[^ ^11^ ^]^ which are associated with a higher risk of EMs.^[^ ^12^ ^]^ Endogenous creatine synthesis primarily occurs through the kidney‐liver axis, with glycine amidinotransferase (GATM) catalyzing guanidinoacetate (GAA) formation, subsequently converted to creatine in hepatocytes by guanidinoacetate N‐methyltransferase (GAMT).^[^ ^13^ ^]^ Creatine is transported into the bloodstream and taken up by cells via the creatine transporter solute carrier family 6 member 8 (SLC6A8). GATM, the rate‐limiting enzyme in creatine synthesis, is upregulated in cancer cells, which correlates with increased creatine metabolism.^[^ ^14^ ^]^ Creatine reportedly facilitates cancer progression, invasion, and metastasis. Creatine levels are significantly elevated in many cancer types and are associated with rapid cancer progression.^[^ ^14^ ^]^ Additionally, creatine protects cells from oxidative stress by reducing mitochondrial activity and decreasing the buildup of intracellular reactive oxygen species (ROS), thus preserving cellular redox homeostasis.^[^ ^15^ ^]^ Considering the tumor‐like characteristics of endometriosis, we hypothesized that creatine may play a vital role in its initiation and progression. However, the precise characteristics and mechanisms of creatine metabolism in the ectopic lesion microenvironment are yet to be fully understood.

Prion protein (PrP, gene name *PRNP*), widely expressed across all cells, influences cellular iron uptake and homeostasis.^[^ ^16^ ^]^ ZRT/IRT‐like protein 14 (ZIP14), with the gene name solute carrier family 39 member 14 (*SLC39A14*), is a divalent metal transporter involved in cellular iron, zinc, and manganese uptake. PrP facilitates the conversion of trivalent iron (Fe^3+^) to divalent iron (Fe^2+^), serving as a ferrireductase (FR) partner of ZIP14.^[^ ^16^ ^]^ Abnormal expression or function of PrP or ZIP14 can elevate intracellular iron and ROS levels, leading to lipid peroxidation and ferroptosis.^[^ ^17^ ^]^ An imbalance in iron homeostasis may reportedly facilitate the implantation and growth of ectopic lesions in endometriosis.^[^ ^18^ ^]^ PrP overexpression in aggressive cancers confers resistance to ferroptosis, promoting resistance to oxaliplatin in colorectal cancer.^[^ ^19^ ^]^ However, the role of PrP in promoting EMs through iron metabolism regulation remains to be determined.

In this study, we aimed to investigate the metabolic characteristics of creatine within ectopic lesions, identify intracellular creatine‐sensing proteins, elucidate the molecular mechanisms of creatine in regulating ferroptosis in endometrial stromal cells, explore metabolic regulation mechanisms in endometriosis, and identify potential therapeutic targets for treatment.

## 2 Results

### 2.1 Creatine Enrichment and Creatine Metabolism Disorders in the Endometriotic Milieu

**Figure 1:**
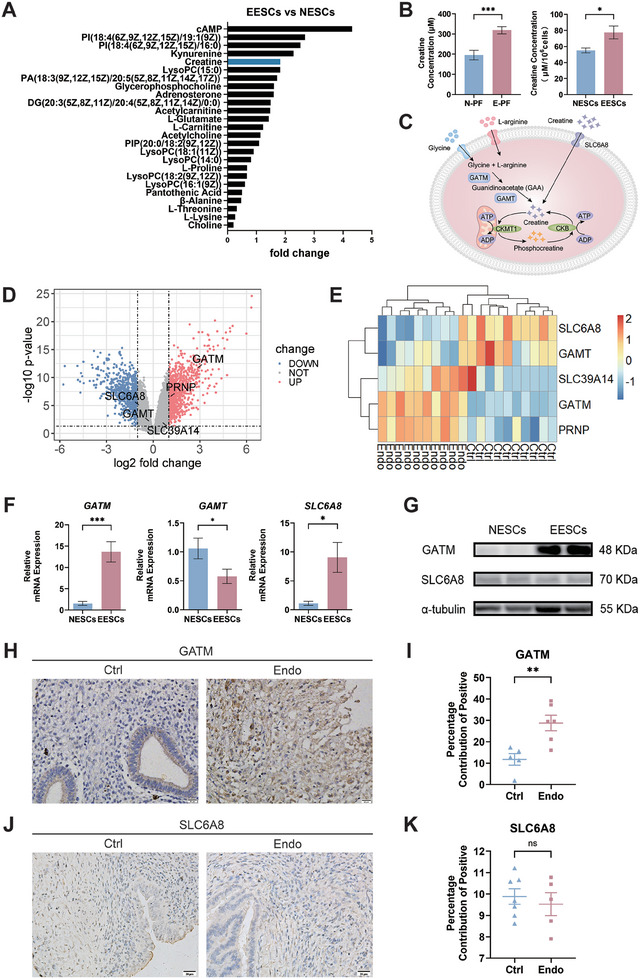
Elevated creatine levels in peritoneal fluid and ectopic endometrial stromal cells (EESCs) of endometriosis patients attributed to endogenous synthesis. A) Visualization of the untargeted metabolomics analysis performed on EESCs and normal endometrial stromal cells (NESCs). Metabolites meeting the criteria of variable importance in projection (VIP) > 1 and *p *< 0.05 were arranged according to the fold change. B) Creatine content in the peritoneal fluid (*n *= 9 for normal; *n *= 20 for endometriosis) and ESCs (*n *= 6 for NESCs; *n *= 7 for EESCs) of control and endometriosis (EMs) groups was measured by a creatine detection kit. C) A schematic diagram of creatine metabolism in target cells. L‐arginine and glycine are used by glycine amidinotransferase (GATM) to synthesize the creatine precursor guanidinoacetate (GAA), which is catalyzed by guanidinoacetate N‐methyltransferase (GAMT) to synthesize creatine. Exogenous creatine relies on the creatine transporter solute carrier family 6 member 8 (SLC6A8). D) The volcano plot illustrating differentially expressed genes (DEGs) from the GSE7305 dataset. Red denotes upregulated genes (*p* < 0.05 and Log2 fold change > 1), while blue denotes downregulated genes (*p* < 0.05 and Log2 fold change < –1). Creatine‐metabolism‐related molecules were highlighted in the plot. E) The heatmap plot displaying gene expression differences in creatine‐metabolism‐related molecules between ectopic tissue from patients with EMs and endometrial tissue from the control groups in the GSE7305 dataset. Red represents relatively upregulated genes, and blue represents relatively downregulated genes. F) Expression levels of creatine‐metabolism‐related molecules in ESCs were assessed using quantitative reverse transcription‐polymerase chain reaction (qRT‐PCR) (*n *= 10 for NESCs and EESCs). G) Representative western blots of creatine‐metabolism‐related molecules in endometrial stromal cells (ESCs). H, J) The expression of GATM or SLC6A8 in normal endometrium (Ctrl) and ectopic lesions (Endo) was assessed via immunohistochemistry. Scale bars, 20 µm. Magnification, 400 ×. I, K) The positive expression areas of GATM (*n *= 5 for Ctrl; *n *= 6 for Endo) or SLC6A8 (*n *= 7 for Ctrl; *n *= 5 for Endo) were quantified using ImageJ. Data in (B, F) are presented as the mean ± standard error of the mean (SEM), *p < 0.05, ***p < 0.001 by two‐tailed Student's *t*‐test with Welch's correction. Data in (I, K) are presented as the mean ± SEM, **p < 0.01, ns, no significant difference by two‐tailed Student's *t*‐test.

We collected endometrial tissue and peritoneal fluid from the control group (without EMs) and ectopic endometrial tissue and peritoneal fluid from the EMs group. We performed untargeted metabolomic analysis on endometrial stromal cells (ESCs) from both groups. Results from the metabolomic analysis showed that creatine increased by a fold change of 1.829, ranking fifth among metabolites that met the criteria of variable importance in projection (VIP) > 1 and *p *< 0.05 (**Figure** **1**A; Tables S2 and S3, Supporting Information). Creatine levels in the peritoneal fluid and EESCs were significantly higher in the EMs group than in the control group (*p *< 0.001, *p *< 0.05; Figure 1B).

In target cells, endogenous creatine synthesis depends on GATM and GAMT, especially GATM. GATM, the rate‐limiting enzyme in creatine synthesis, uses L‐arginine and glycine to synthesize the creatine precursor, GAA, which GAMT subsequently catalyzes to produce creatine.^[^ ^20^ ^]^ The uptake of exogenous creatine is facilitated by the creatine transporter SLC6A8 (Figure 1C). We analyzed differences in gene expression between EMs ectopic tissue and control endometrium tissue using the GEO database, focusing on annotating creatine‐metabolism‐related molecules. Analysis of the GSE7305 dataset revealed that expression ratios of *GATM* and *PRNP* between ectopic tissue from patients with EMs and endometrial tissue from the control groups were > 1 (*p* < 0.05). Conversely, the levels of *SLC6A8*, *GAMT*, and *SLC39A14* did not significantly differ (Figure 1D,E).

We performed quantitative reverse transcription‐polymerase chain reaction (qRT‐PCR) and western blotting (WB) to validate the expression of creatine‐metabolism‐related molecules in ESCs. *SLC6A8* and *GATM* mRNA expression levels were significantly higher in EESCs than in normal endometrial stromal cells (NESCs) (Figure 1F). Western blot analysis indicated significantly elevated GATM protein expression in EESCs, whereas SLC6A8 protein expression showed no significant change (Figure 1G). Immunohistochemistry (IHC) staining indicated significantly higher GATM expression levels in ectopic lesions than in control endometrium (*p* < 0.01), with no significant change observed in SLC6A8 expression levels (*p* > 0.05; Figure 1H–K). In conclusion, an abnormality in creatine metabolism occurs in ectopic lesions of patients with EMs. Increased creatine content in the peritoneal fluid and EESCs, as well as creatine accumulation in ESCs, are attributable to endogenous synthesis.

### 2.2 Creatine Promotes Ferroptosis Resistance and Enhances Human Endometrial Stromal Cell Viability in an Ectopic Iron‐Rich Environment

Creatine reportedly plays a vital role in cancer progression, invasion, and metastasis. Given the similar pathological behaviors of EMs and cancers,^[^ ^9^ ^]^ the aberrant expression profile of creatine metabolism in endometriotic lesions led us to explore the impact of creatine on the proliferation, migration, invasion, and apoptosis of ESCs, as well as its role in an ectopic iron‐overload environment. After exposing human endometrial stromal cells (HESCs) to a range of creatine concentrations from 0 to 1000 µM for 24 h, we assessed the effects of a high creatine environment on HESCs migration using the wound‐healing assay and evaluated invasion capabilities using transwell chambers. Creatine showed no significant effect on HESCs migration or invasion (*p* > 0.05; Figure S1A–D, Supporting Information). After treatment with creatine concentrations ranging from 0 to 1000 µM for 24 or 48 h, we examined the effects of creatine accumulation on HESCs proliferation using cell counting kit‐8 (CCK‐8) assays, which revealed no significant changes in the viability of HESCs (*p *> 0.05; Figure S1E,F, Supporting Information).

**Figure 2:**
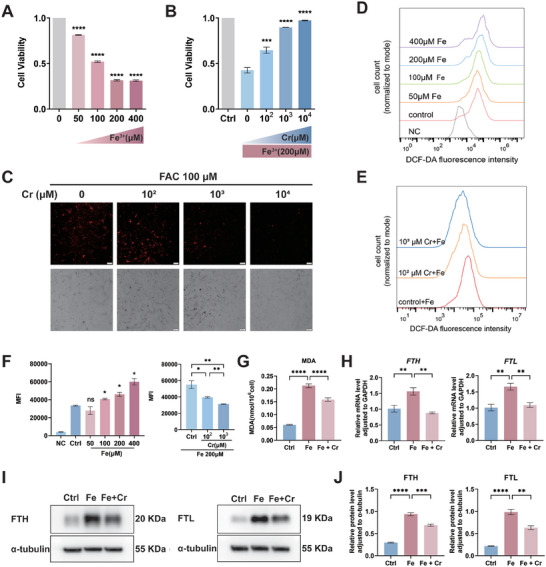
Creatine reduces cellular iron content in ESCs and enhances cell viability in an ectopic iron‐overload environment. A) Cell counting kit‐8 (CCK‐8) assays were used to assess cell viability in cells treated with 50, 100, 200, and 400 µM Fe^3+^ for 24 h. Cell viability decreased with increasing Fe^3+^ concentration (*n *= 3). B) After treating human endometrial stromal cells (HESCs) with 0, 100, 1000, and 10000 µM creatine media and 200 µM Fe^3+^ for 24 h, cell viability increased as the creatine concentrations increased (*n *= 3). C) Following treatment of HESCs with 0, 100, 1000, and 10000 µM creatine media in the presence of 100 µM Fe^3+^, RhoNox‐1, a fluorescent probe for the specific detection of divalent iron, was used to detect free divalent iron ions in HESCs. Scale bars, 200 µm. Magnification, 100 ×. D) Intracellular reactive oxygen species (ROS) levels were measured by flow cytometry in HESCs treated with 0, 50, 100, 200, and 400 µM Fe^3+^. ROS concentration in HESCs increased with increasing iron concentrations. E) At 200 µM iron, ROS concentration in HESCs decreased with increasing creatine concentrations. F) Results of flow cytometry from (D) and (E) were calculated and presented as the mean fluorescence intensity (MFI) (*n *= 3). HESCs were divided into 3 groups: control, treated with 200 µM iron, and treated with 200 µM iron and 1 mM creatine. G) The malondialdehyde (MDA) detection kit was used to detect the level of superoxide lipids in HESCs (*n *= 3). H) The mRNA expression levels of ferritin heavy chain (*FTH*) and ferritin light chain (*FTL*), in HESCs were evaluated by qRT‐PCR. I) Representative western blots of FTH and FTL in HESCs. J) Densitometry analysis of protein expression levels was quantified using ImageJ (*n *= 3). Data in (A, B, F−H, J) are presented as the mean ± SEM, *p < 0.05, **p < 0.01, ***p < 0.001, ****p < 0.0001, ns, no significant difference by one‐way analysis of variance (ANOVA) test.

An iron‐rich environment in ectopic lesions and peritoneal fluid results from the accumulation of retrograde menstrual debris and repeated bleeding episodes, as reported in patients with EMs.^[^ ^6^ ^]^ However, EESCs in EMs reportedly resist iron‐mediated ferroptosis and survive in ectopic high‐iron lesions,^[^ ^10^ ^]^ though the underlying mechanism remains unclear. To detect cell viability, we treated HESCs with different iron concentrations for 24 h. Within the tested range of iron concentrations, a decrease in cell viability was observed as the concentration increased (*p *< 0.0001), with the cell viability at 200 µM and 400 µM iron concentrations appearing similar (**Figure** **2**A). Consequently, 200 µM was chosen as the subsequent iron treatment concentration for HESCs.

Subsequently, we investigated the effect of creatine on HESCs viability in a high‐iron environment by treating the cells with various creatine concentrations for 24 h at the same iron concentration. At an iron concentration of 200 µM, cell viability increased as the creatine concentration increased (*p *< 0.001; Figure 2B). We hypothesized that creatine counteracts the decline in cell viability induced by high iron levels, potentially rescuing HESCs from ferroptosis in a high‐iron environment. Following treatment with 100 µM iron and varying concentrations of creatine for 24 h, RhoNox‐1, a fluorescent probe for the specific detection of divalent iron, was used to detect free divalent iron ions in HESCs. In the presence of 100 µM iron, HESCs showed a decrease in intracellular free divalent iron levels as the creatine concentration increased (Figure 2C).

We further explored the effects of creatine on oxidative stress and lipid peroxidation resulting from iron overload. 2′,7′‐Dichlorodihydrofluorescein diacetate (DCFH‐DA) was used to detect cellular ROS accumulation in HESCs, which indicated an increase in ROS concentration as the iron concentration increased (*p *< 0.05; Figure 2D,F). Additionally, at 200 µM iron, the ROS concentration in HESCs decreased as the creatine concentration increased (*p *< 0.05; Figure 2E,F). Malondialdehyde (MDA) is the end product of ferroptosis. We observed that creatine inhibited the iron‐induced upregulation of MDA content in HESCs after incubation for 24 h (*p *< 0.0001; Figure 2G). Given the critical balance between free divalent iron ions and iron storage forms, we measured the levels of ferritin heavy chain (FTH) and ferritin light chain (FTL), which play important roles in maintaining iron homeostasis.^[^ ^21^ ^]^ HESCs were divided into 3 groups: control, iron‐treated, and creatine + iron‐treated. qRT‐PCR and western blotting revealed significant differences in FTH and FTL expression levels between the iron‐ and creatine + iron‐treated groups (Figure 2H–J). Collectively, these findings indicate that iron overload leads to an increase in both free divalent iron ions and stored iron, along with the accumulation of ROS and MDA, and a decrease in cell viability. However, creatine treatment significantly reduced the cellular iron concentrations, mitigated oxidative stress and lipid peroxidation, and prevented iron‐induced ferroptosis.

### 2.3 PrP as a Potential Binding Protein of Creatine, with Enhanced Expression in EMs

**Figure 3:**
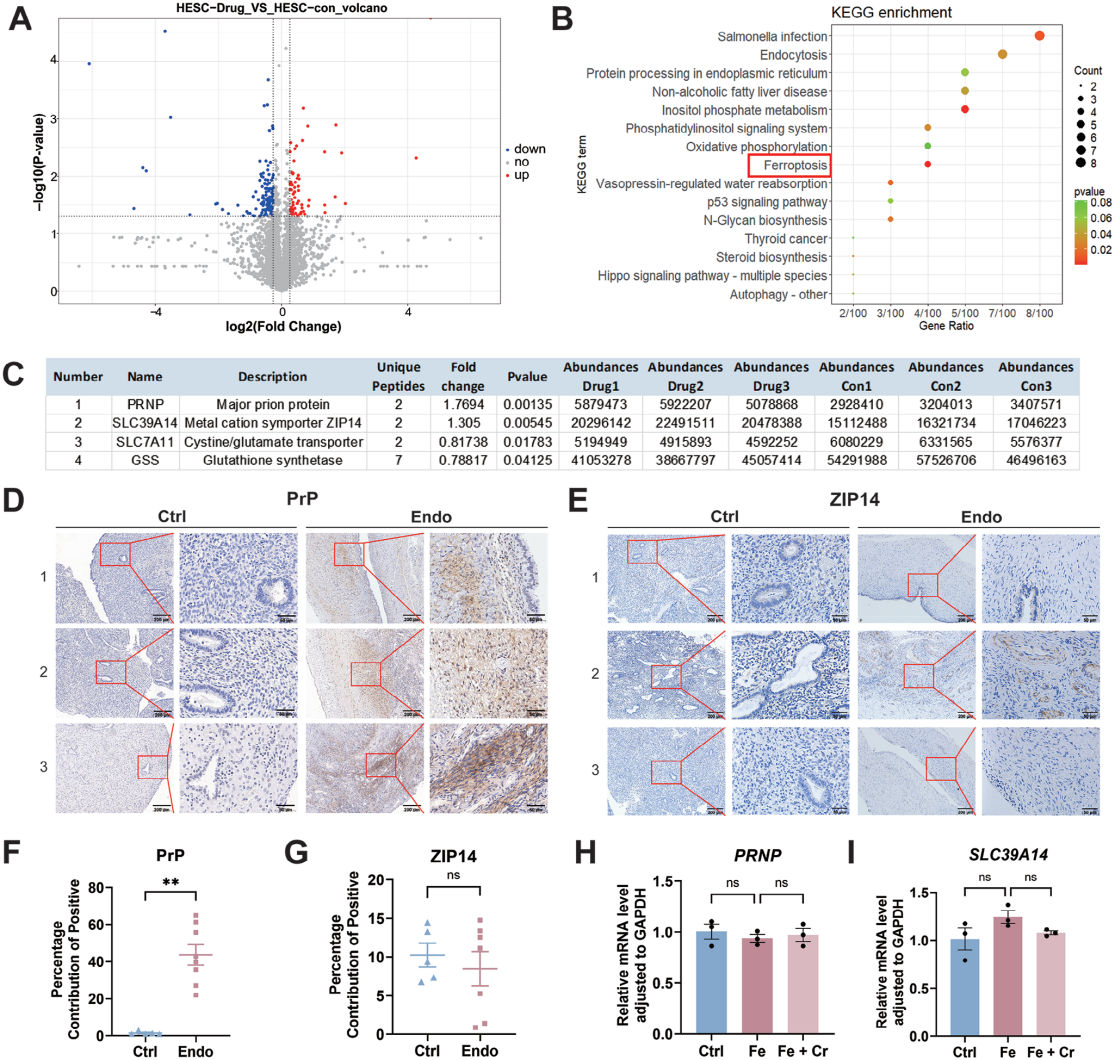
Prion protein (PrP) may be a potential binding protein for creatine. Untreated HESCs were lysed and subjected to the drug affinity‐responsive target stabilization (DARTS) assay and mass spectrometry analysis. A) Volcano plots illustrating differentially expressed proteins between creatine and control groups. Red denotes 65 upregulated proteins (fold change ≥ 1.2 and *p* < 0.05), while blue denotes 119 downregulated proteins (fold change ≤ 0.8 and *p* < 0.05). B) Kyoto Encyclopedia of Genes and Genomes (KEGG) pathway enrichment analysis for the differentially expressed proteins identified in (A). C) Four candidate proteins were identified in the “ferroptosis” pathway from the KEGG enrichment analysis. D) The expression levels of PrP (gene name *PRNP*) and ZRT/IRT‐like protein 14 (ZIP14, gene name *SLC39A14*) in normal endometrium and ectopic lesions were measured using immunohistochemistry. Scale bars, 200 µm and 50 µm. Magnification, 100 × and 400 ×. F, G) The positive expression areas of (D) (*n *= 5 for Ctrl, *n *= 8 for Endo) and (E) (*n *= 5 for Ctrl, *n *= 7 for Endo) were quantified with ImageJ. H, I) The expression levels of *PRNP* and *SLC39A14* in HESCs were evaluated by qRT‐PCR (*n *= 3). Data in (F, G) are presented as the mean ± SEM, **p < 0.01, ns, no significant difference by two‐tailed Student's *t*‐test. Data in (H, I) are presented as the mean ± SEM, ns, no significant difference by one‐way ANOVA test.

We employed drug affinity‐responsive target stabilization assay (DARTS) technology and mass spectrometry to elucidate the molecular mechanisms underlying the impact of creatine on ferroptosis resistance and enhancement of cell viability in HESCs. Volcano plots revealed that the expression levels of 65 proteins were upregulated (fold change ≥ 1.2 and *p* < 0.05), whereas those of 119 proteins were downregulated (fold change ≤ 0.8 and *p* < 0.05; **Figure** **3**A). DARTS identifies ligand‐bound targets in lysates based on their increased resistance to proteolysis.^[^ ^22^ ^]^ Therefore, these 65 upregulated proteins were identified as potential creatine targets (Table S5, Supporting Information). To elucidate the pathways and molecular functions associated with the proteins previously identified as differentially expressed, we conducted Kyoto Encyclopedia of Genes and Genomes (KEGG) enrichment analyses. The differentially expressed proteins in creatine groups participated in pathways such as “Salmonella infection,” “Endocytosis,” “Inositol phosphate metabolism,” and notably, “Ferroptosis” (Figure 3B). Within the “Ferroptosis” pathway, 2 proteins, PrP and ZIP14, were significantly upregulated and selected for further investigation (Figure 3C).

We performed IHC staining to examine the expression of PrP and ZIP14 in normal endometrial tissues and ectopic lesions, revealing that PrP expression was significantly higher in ectopic lesions than in control tissues (*p *< 0.01; Figure 3D,F). Conversely, ZIP14 demonstrated low expression levels, with no significant difference between ectopic and normal tissues (*p *> 0.05; Figure 3E,G). Considering the pivotal roles of PrP and ZIP14 in cellular iron uptake and homeostasis,^[^ ^23^ ^]^ we explored the potential impact of creatine on the expression of these proteins and their effect on the development of ferroptosis resistance. We divided HESCs into 3 groups: control (Ctrl), iron‐treated (Fe), and creatine + iron‐treated (Fe + Cr). qRT‐PCR revealed no significant differences in *PRNP* and *SLC39A14* expression between the Fe and Fe + Cr groups (*p *> 0.05; Figure 3H,I). These findings led us to hypothesize that creatine promotes high iron tolerance in HESCs by binding to PrP and interfering with its function rather than by altering its expression.

### 2.4 Creatine Inhibits Ferroptosis Triggered by *PRNP* Overexpression in HESCs

**Figure 4:**
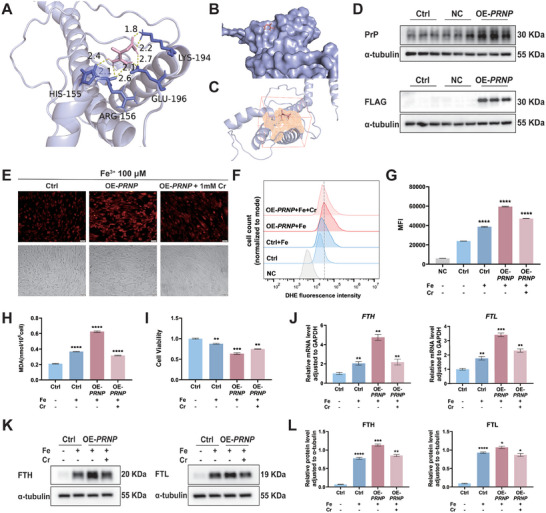
Creatine inhibits ferroptosis induced by *PRNP* overexpression in HESCs. A,B) Possible docked conformations for creatine (pink) bound to PrP (purple). C) Creatine binds to the active center (orange) of PrP. D) Representative western blots of *PRNP* in HESCs after transfecting with *PRNP*‐overexpressing lentiviruses for 72 h. The control and *PRNP*‐overexpressing HESCs were divided into 4 subgroups: HESCs treated with phosphate‐buffered saline (PBS) (Ctrl), HESCs treated with 200 µM iron (Ctrl + Fe), OE‐*PRNP* HESCs treated with 200 µM iron (OE‐*PRNP* + Fe), and OE‐*PRNP* HESCs treated with 200 µM iron and 1 mM creatine (OE‐*PRNP* + Fe + Cr) for 24 h. E) RhoNox‐1 was used to detect the levels of free divalent iron ions in the above 4 groups in the presence of 100 µM Fe^3+^. Scale bars, 50 µm. Magnification, 200 ×. F) Intracellular ROS levels were measured by flow cytometry in the 4 groups. G) Results of flow cytometry from (F) were calculated and presented as MFI (*n *= 3). H) The MDA detection kit was used to detect the levels of superoxide lipids in the 4 groups (*n *= 6). I) CCK‐8 assays were used to measure the cell viability of HESCs (*n *= 3). J) *FTH* and *FTL* mRNA expression levels in HESCs were evaluated by qRT‐PCR (*n *= 3). K) Representative western blots of FTH and FTL in the 4 groups. L) Densitometry analysis was used to quantify protein expression levels by ImageJ (*n *= 3). Data in (G−J, L) are presented as the mean ± SEM, *p < 0.05, **p < 0.01, ***p < 0.001, ****p < 0.0001 by one‐way ANOVA test.

Utilizing DARTS results, we used Autoock software^[^ ^24^ ^]^ to generate docked conformations of creatine bound to PrP, and visualized potential structural models with PyMOL software.^[^ ^25^ ^]^ We hypothesized that creatine binds to the 4 amino acid subunits of PrP via hydrogen bonding (**Figure** **4**A,B). The DoGSiteScorer tool within the Proteins Plus platform was employed to predict the active site of PrP.^[^ ^26^ ^]^ Surprisingly, our findings indicated that creatine binds to the active site of PrP, potentially inhibiting its activity (Figure 4C). Since creatine did not influence PrP expression, we investigated its impact on PrP function. PrP, acting as a ferrireductase on cell surfaces and endosomes, catalyzes the conversion of trivalent iron (Fe^3+^) to divalent iron (Fe^2+^). Dysfunction of PrP contributes significantly to iron imbalance.^[^ ^27^ ^]^ Hence, we transfected cells with *PRNP‐*overexpressing lentiviruses and selected them using 10 mg L^−1^ puromycin to obtain stably transfected HESCs that overexpress *PRNP*. Fluorescent imaging, qRT‐PCR, and western blotting confirmed successful transfection and elevated *PRNP* expression in HESCs (Figure 4D; Figure S2, Supporting Information). Untreated HESCs were divided into 2 subgroups: one treated with phosphate‐buffered saline (PBS) (Ctrl) and the other with 200 µM iron (Ctrl + Fe). Simultaneously, *PRNP*‐overexpressing HESCs were randomly divided into 2 subgroups: one treated with 200 µM iron (OE‐*PRNP* + Fe) and the other with 200 µM iron and 1 mM creatine (OE‐*PRNP* + Fe + Cr). After 24 h of incubation, subsequent experiments were performed on these 4 groups.

In the presence of 100 µM Fe^3+^, RhoNox‐1 indicated that intracellular free divalent iron levels increased in the OE‐*PRNP* + Fe group and decreased following creatine incubation (Figure 2E). Dihydroethidium (DHE), with red fluorescence, was used to detect cellular ROS accumulation, showing that upregulated *PRNP* expression led to a significant increase in ROS concentration compared to the Ctrl + Fe group. Notably, the addition of creatine to the OE‐*PRNP* + Fe + Cr group resulted in a decrease in ROS concentration (*p *< 0.0001; Figure 4F,G). Compared with that in the Ctrl + Fe group, MDA levels were elevated in the OE‐*PRNP* + Fe group but decreased with the addition of creatine (*p *< 0.0001; Figure 4H). Correspondingly, cell viability decreased in the OE‐*PRNP* + Fe groups (*p *< 0.001) and recovered to a certain extent after creatine incubation (*p *< 0.01; Figure 4I). Additionally, qRT‐PCR and western blotting revealed a significant increase in FTH and FTL expression levels in the OE‐*PRNP* + Fe group compared to the Ctrl + Fe group (*p *< 0.01, *p *< 0.001) and a significant decrease after creatine incubation (*p *< 0.01, *p *< 0.05; Figure 4J–L). Given these results, we determined that *PRNP* overexpression increased the concentrations of both intracellular free divalent iron and storage iron, as well as levels of ROS and MDA, thereby inducing ferroptosis and leading to a reduction in cell viability. Creatine counteracted these effects by decreasing intracellular iron concentration, inhibiting oxidative stress and lipid peroxidation, and enhancing cell viability. Therefore, we hypothesized that creatine supplementation inhibits PrP function, thereby rescuing HESCs from ferroptosis.

### 2.5 Silencing *PRNP* Protects Cells from Ferroptosis Without Additional Benefit from Creatine

**Figure 5:**
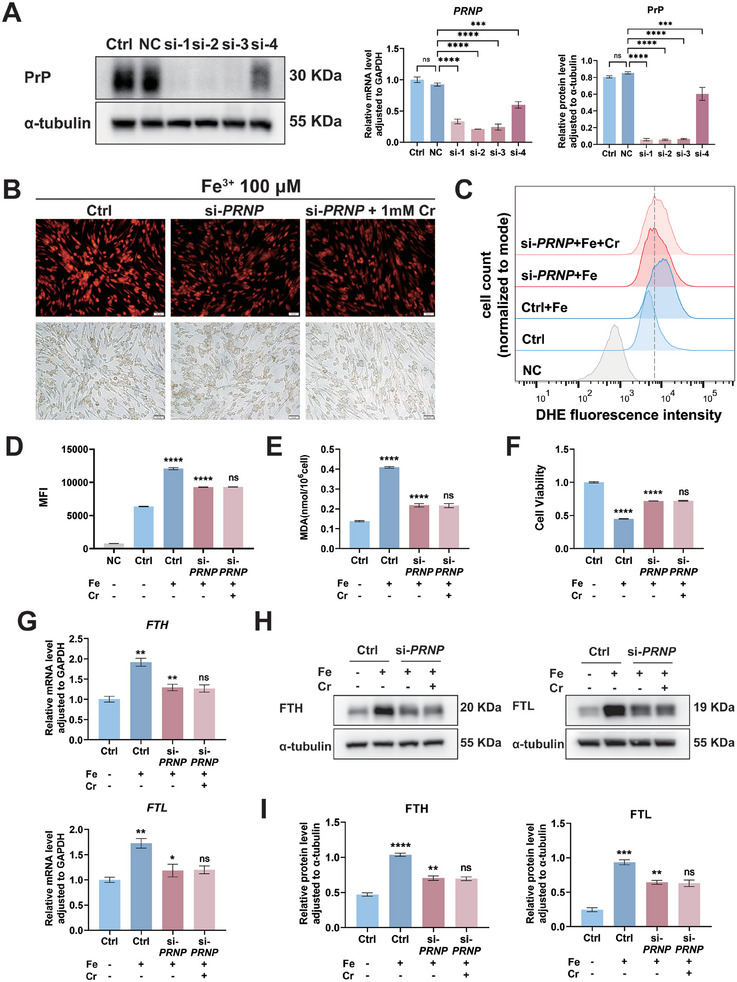
Creatine failed to further reduce cellular iron concentration and enhance cell viability in si‐*PRNP* HESCs. A) Representative western blots and qRT‐PCR results of PrP expression levels in HESCs after transfection with *PRNP*‐474 (si‐1), *PRNP*‐574 (si‐2), *PRNP*‐674 (si‐3), and *PRNP*‐2574 (si‐4) siRNA oligos for 48 h. Densitometry analysis for protein expression levels from WB was quantified using ImageJ (*n *= 3). Untreated HESCs and si‐*PRNP* HESCs were divided into 4 groups: HESCs treated with PBS (Ctrl), HESCs treated with 200 µM iron (Ctrl + Fe), si‐*PRNP* HESCs treated with 200 µM iron (si‐*PRNP* + Fe), and si‐*PRNP* HESCs treated with 200 µM iron and 1 mM creatine (si‐*PRNP* + Fe + Cr). B) RhoNox‐1 was used to detect the levels of free divalent iron ions in the above 4 groups in the presence of 100 µM Fe^3+^. Scale bars, 50 µm. Magnification, 200 ×. C) Intracellular ROS levels were measured by flow cytometry in 4 groups. D) Results of flow cytometry from (C) were calculated and presented as MFI (*n *= 3). E) The MDA detection kit was used to detect the level of superoxide lipid in 4 groups (*n *= 5). F) CCK‐8 assays were performed to measure the cell viability in HESCs (*n *= 3). G) *FTH* and *FTL* mRNA expression levels in HESCs were assessed using qRT‐PCR (*n *= 3). H) Representative western blots of FTH and FTL in 4 groups. I) Densitometry analysis of protein expression levels was quantified using ImageJ (*n *= 3). Data in (A, D−G, I) are presented as the mean ± SEM, *p < 0.05, **p < 0.01, ***p < 0.001, ****p < 0.0001, ns, no significant difference by one‐way ANOVA test.

To determine whether creatine induces ferroptosis resistance in HESCs by targeting PrP, we transfected HESCs with small interfering RNA (siRNA) oligos to downregulate *PRNP* expression. qRT‐PCR and western blotting revealed decreased PrP expression levels in HESCs after transfection with *PRNP*‐724 (si‐1), *PRNP*‐597 (si‐2), *PRNP*‐476 (si‐3), and *PRNP*‐2407 (si‐4), with *PRNP*‐597 (si‐2) selected for subsequent experiments (*p *< 0.0001; **Figure** **5**A). Untreated HESCs were divided into 2 subgroups: one treated with PBS (Ctrl) and the other with 200 µM iron (Ctrl + Fe). Meanwhile, si‐*PRNP* HESCs were divided into 2 subgroups: one treated with 200 µM iron (si‐*PRNP* + Fe) and the other with 200 µM iron plus 1 mM creatine (si‐*PRNP* + Fe + Cr). In the presence of 100 µM Fe^3+^, RhoNox‐1 indicated that intracellular free divalent iron levels were diminished in the si‐*PRNP* + Fe groups, with no additional decrease following creatine incubation (Figure 5B). ROS assessment via DHE demonstrated that reduced *PRNP* expression in the si‐*PRNP* + Fe group led to a significant decrease in ROS levels relative to the Ctrl + Fe group (*p *< 0.0001). However, no significant difference in ROS levels was observed between the si‐*PRNP* + Fe and si‐*PRNP* + Fe + Cr groups (*p *> 0.05; Figure 5C,D). Upon exposure to 200 µM iron, MDA levels decreased in the si‐*PRNP* + Fe group relative to the Ctrl + Fe group. Similarly, no significant difference in MDA levels was observed between the si‐*PRNP* + Fe and si‐*PRNP* + Fe + Cr groups (*p *< 0.0001, *p* > 0.05; Figure 5E). Correspondingly, the CCK‐8 assays revealed increased cell viability in the si‐*PRNP* + Fe group (*p *< 0.0001). Following creatine incubation, there was no further increase in cell viability of HESCs (*p* > 0.05; Figure 5F). Additionally, qRT‐PCR and western blotting revealed a significant decrease in FTH and FTL expression levels between the Ctrl + Fe and si‐*PRNP* + Fe groups (*p *< 0.01, *p *< 0.05), with no significant changes after creatine incubation ((*p* > 0.05; Figure 5G–I). These results suggested that *PRNP* silencing protected cells from ferroptosis, whereas creatine did not further reduce cellular iron concentrations in si‐*PRNP* HESCs, indicating that creatine induces tolerance to high‐iron environments by inhibiting PrP activity.

### 2.6 Creatine Facilitates the Proliferation of Ectopic Lesions by Suppressing PrP in Mouse Models of EMs

**Figure 6:**
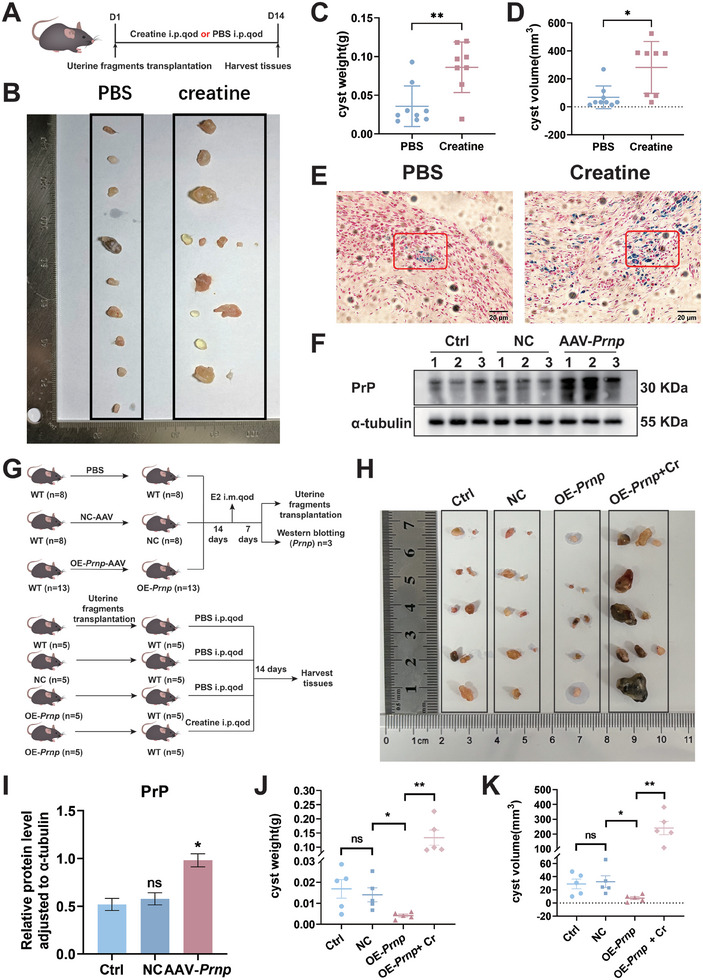
Creatine may promote the development of EMs by inhibiting PrP in mouse models. A) Schematic diagram of the EMs mouse models. B) The number of ectopic lesions was significantly higher in the creatine group (*n *= 8) than in the control group (*n *= 9). C, D) The weight and volume of ectopic lesions were significantly increased in the creatine group (*n *= 9 for control, *n *= 8 for creatine group). E) Prussian blue staining shows iron deposits in ectopic lesions. Scale bars, 20 µm. Magnification, 400 ×. F) Representative western blots of PrP in 3 groups. G) Schematic diagram of AAV‐*Prnp* mouse models, including AAV‐*Prnp* intravenous injection and creatine supplementation via intraperitoneal injection. H) The number of ectopic lesions was lower in the OE‐*Prnp* group (*n *= 5) than that in the NC group (*n *= 5), whereas it was higher in the OE‐*Prnp* + Cr group (*n *= 5) than that in the OE‐*Prnp* group. I) Densitometry analysis of protein expression levels in (F) was quantified using ImageJ (*n *= 3). J, K) Both the weight and volume of ectopic lesions decreased in the OE‐*Prnp* group and subsequently increased following creatine supplementation (*n *= 5). Data in (C) are presented as the mean ± SEM, **p < 0.01 by two‐tailed Student's *t*‐test. Data in (D) are presented as the mean ± SEM, *p < 0.05 by two‐tailed Student's *t*‐test with Welch's correction. Data in (I−K) are presented as the mean ± SEM, *p < 0.05, **p < 0.01 by one‐way ANOVA test.

To validate the impact of creatine on the development and establishment of ectopic lesions, we established a mouse model of EMs by intraperitoneal injection of endometrial fragments, as described previously.^[^ ^28^ ^]^ Allografts were utilized to construct the mouse models. Four days postsurgery, we randomly assigned the recipient mice into 2 groups. The control group (*n* = 5) received an intraperitoneal injection of PBS solution every 3 days, while the creatine group (*n* = 5) received a creatine solution (13 mg mL^−1^, 100 mM) on the same schedule (**Figure** **6**A). After 14 days of treatment, we euthanized the mice were euthanized by cervical dislocation and harvested the ectopic lesions. As anticipated, the creatine group exhibited a higher number of ectopic lesions than that of the control group (Figure 6B), with a significant increase in both the weight and volume of these lesions (*p *< 0.01, *p *< 0.05; Figure 6C,D). We then fixed, paraffin‐embedded, and stained a portion of the tissue from the ectopic lesions with Prussian blue to highlight iron deposits. (Figure 6E).

To determine whether creatine promotes EMs development by inhibiting PrP (gene name *Prnp* in *Mus musculus*), we developed an adeno‐associated virus 9 (AAV 9)‐*Prnp* mouse model. We divided the mice into 3 groups: control (*n* = 8), negative control (NC; *n* = 8), and *Prnp* overexpression (OE‐*Prnp*) (*n* = 13), which received intravenous injections of PBS, negative control adeno‐associated viruses (AAVs), and *Prnp*‐overexpression AAVs, respectively (Figure 6G). Three weeks later, we euthanized 3 mice from each group, collected endometrial tissues, and performed western blotting. *Prnp* expression was significantly higher in the OE‐*Prnp* group than in the NC group (*p* < 0.05), with no significant difference between the control and NC groups (*p* > 0.05; Figure 6F,I). We then divided an additional 20 recipient mice into 3 groups (*n* = 5, 5, and 10) and intraperitoneally injected them with the remaining endometrial tissue fragments from donor mice at a 1:1 ratio for each corresponding group. Starting on day 4, the control and NC groups received intraperitoneal PBS injections once every 3 days. Simultaneously, we divided OE‐*Prnp* group into 2 subgroups (*n* = 5) on day 4. One subgroup continued to receive PBS (OE‐*Prnp* group), whereas the other received creatine solution (13 mg mL^−1^, 100 mM) via intraperitoneal injection once every 3 days (OE‐*Prnp* +Cr group) (Figure 6G). After another 2 weeks, we euthanized the mice and collected and analyzed the ectopic lesion tissues. The OE‐*Prnp* group exhibited a reduction in the number of ectopic lesions compared to the NC group, whereas the OE‐*Prnp* + Cr group showed an increase relative to the OE‐*Prnp* group (Figure 6H). Furthermore, both the weight and volume of the ectopic lesions decreased in the OE‐*Prnp* group and increased following creatine supplementation (*p *< 0.01, *p *< 0.01; Figure 6J,K).

These findings suggest that creatine potentially promotes EMs development. Additionally, *Prnp* overexpression reduced the number of ectopic endometrial lesions, whereas creatine supplementation stimulated the implantation and growth of these lesions. Therefore, we hypothesize that creatine accelerates the development of EMs by inhibiting the function of PrP in mouse models.

**Figure 7:**
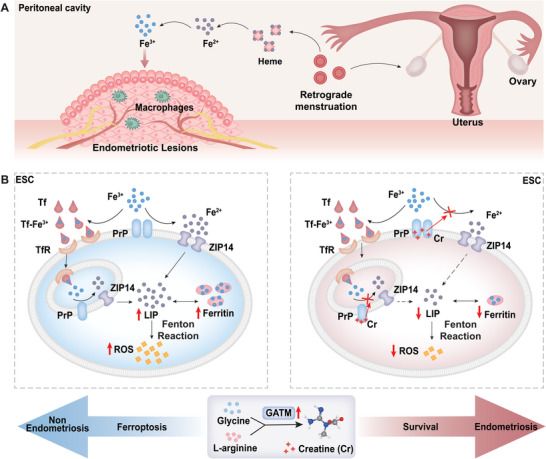
Schematic diagram of the mechanism underlying the impact of creatine on ferroptosis resistance in ESCs through PrP inhibition. A) Retrograde menstruation through the fallopian tubes leads to the implantation and growth of endometriotic lesions in the peritoneum and pelvic organs. The erythrocyte lysis releases hemoglobin and its byproducts, heme, and iron, which serve as the source of iron overload in the peritoneal cavity of women with EMs. B) Extracellular iron primarily exists in the form of trivalent iron (Fe^3+^), which is internalized via the TfR pathway by binding to Tf. Upon extracellular iron overload, excess Fe^3+^ is reduced to divalent iron (Fe^2+^) by PrP and transported through ZIP14. Additionally, Fe^3+^ released from Tf within acidic endosomes is reduced by PrP and transported to the cytosol through ZIP14. Excess iron is stored in the form of ferritin. Fe^2+^ in the LIP catalyzes the Fenton reaction, resulting in the generation of ROS, lipid peroxidation, and ferroptosis, thereby preventing the occurrence and progression of endometriosis. When ESCs express GATM at high levels and accumulate creatine, creatine binds to the active canter of PrP. This interaction inhibits its enzymatic activity, interrupts the reduction process of Fe^3+^ to Fe^2+^, and reduces iron uptake. Consequently, a decrease in the concentration of Fe^2+^ in the intracellular LIP leads to a reduction in ROS and lipid hydroperoxide levels, thus preventing ferroptosis in ESCs and contributing to the onset and growth of endometriosis. Cr, creatine; PrP, prion protein; ZIP14, ZRT/IRT‐like protein 14; Tf, transferrin; TfR, transferrin receptor; LIP, labile iron pool; ROS, reactive oxygen species; ESC, endometrial stromal cell; GATM, glycine amidinotransferase.

## 3 Conclusion

EMs affects ≈10% of women of reproductive age; however, its pathogenesis remains partially understood.^[^ ^29^ ^]^ The widely accepted retrograde menstruation theory proposes that endometrial cells, dislodged during menstruation, pass through the fallopian tubes into the peritoneal cavity, where they implant in the peritoneum and pelvic organs and proliferate.^[^ ^3a^ ^]^ Despite the prevalence of retrograde menstruation, not all women develop EMs, suggesting additional factors contribute to disease onset. The accumulation of menstrual debris and repeated bleeding episodes creates a high‐heme and high‐iron environment in ectopic lesions, conducive to the development of EMs.^[^ ^30^ ^]^ The erythrocyte lysis releases pro‐oxidant hemoglobin byproducts, heme, and iron, contributing to iron overload.^[^ ^4^ ^]^ This iron imbalance promotes the implantation and proliferation of ectopic lesions.^[^ ^18^ ^]^ Iron overload reportedly activates the nuclear factor‐kappaB (NF‐kB) pathway and increases the divalent metal transporter‐1 (DMT1) expression, thereby enhancing oxidative stress and proinflammatory signaling.^[^ ^31^ ^]^ Moreover, iron‐induced nitric oxide (NO) overproduction may impair the apoptosis of peritoneal macrophages, facilitating cellular proliferation within the peritoneal cavity.^[^ ^32^ ^]^ Typically, a high‐iron environment triggers ferroptosis, an iron‐mediated, non‐apoptotic programmed cell death pathway in EESCs.^[^ ^6^ ^]^ However, abnormal EESCs in endometriosis, which are characterized by resistance to ferroptosis, can survive, implant, and establish endometriotic lesions within the high‐iron environment.^[^ ^10^ ^]^ The mechanisms underlying EESCs survival and its role in promoting EMs formation and development remain unclear.

Creatine is a naturally occurring, small‐molecule, nitrogen‐containing organic acid. Untargeted metabolomic analysis performed on NESCs and EESCs suggests that the creatine concentration is elevated in the EESCs of patients with EMs. Moreover, the creatine content in the peritoneal fluid and EESCs was significantly higher in the EMs group than in the control group. Creatine synthesis is a 2‐step enzymatic process involving GATM and GAMT. The rate‐limiting step is catalyzed by GATM, which transfers the amidino group from arginine to glycine, producing GAA and thereby directly influencing creatine production rates.^[^ ^33^ ^]^ Our focus was on GATM due to its pivotal role in creatine synthesis, as evidenced by increased expression at both the mRNA and protein levels in EESCs. IHC staining further confirmed significantly higher GATM expression in ectopic lesions compared to controls. The potential negative feedback mechanism induced by high creatine concentrations on GAMT expression may be a regulatory response to maintain cellular homeostasis. The increase in SLC6A8 mRNA in EESCs, without a corresponding increase in protein levels, could be due to post‐transcriptional modifications that affect mRNA stability, splicing, or translation efficiency, leading to decoupled mRNA and protein expression.^[^ ^34^ ^]^ Collectively, these results suggest that creatine accumulated from endogenous synthesis.

Creatine reportedly plays a crucial role in cancer progression, invasion, and metastasis.^[^ ^14^ ^]^ Although EMs is considered a benign lesion, it shares several characteristics, including proliferation, infiltration, metastasis, and recurrence, with invasive cancers.^[^ ^9^ ^]^ In this study, we explored the role of creatine in the pathogenesis of endometriosis and its possible mechanisms in inducing ferroptosis resistance. However, creatine did not affect the migration, invasion, or viability of HESCs, but, unexpectedly, in an iron‐overloaded environment, creatine could rescue the decline in cell viability induced by high iron levels. These results suggest that iron overload leads to an increase in the levels of free divalent iron ions and stored iron, the accumulation of ROS and MDA, and a decrease in cell viability. Conversely, creatine treatment significantly reduced the cellular iron concentration, inhibited oxidative stress and lipid peroxidation, and rescued cells from iron‐induced ferroptosis.

DARTS technology coupled with mass spectrometry was used to explore the molecular mechanisms by which creatine induces ferroptosis resistance and enhances HESCs cell viability. KEGG enrichment analysis demonstrated that the differentially expressed proteins in groups treated with creatine, including PrP and ZIP14, were involved in the “ferroptosis” pathway. PrP catalyzes the conversion of Fe^3+^ to Fe^2+^ and serves as a ferrireductase partner for ZIP14,^[^ ^23^ ^]^ a divalent metal transporter that mediates cellular iron uptake. Abnormal expression or function of either PrP or ZIP14 leads to the dysregulation of iron homeostasis, culminating in lipid peroxidation and ferroptosis.^[^ ^17^ ^]^ An imbalance in iron homeostasis promotes the establishment and growth of ectopic lesions in endometriosis.^[^ ^18^ ^]^ Additionally, Peng et al. found that high estrogen levels induce augmented expression of PrP in EESCs, which acts as a critical mediator promoting cholesterol accumulation and estrogen production by negatively regulating the peroxisome proliferator‐activated receptor alpha (PPARα) pathway, ultimately promoting the progression EMs.^[^ ^35^ ^]^ Furthermore, *PRNP* is aberrantly expressed in several invasive cancers.^[^ ^36^ ^]^ The RNA‐binding motif single‐stranded interacting protein 1 (RBMS1) reportedly prevents ferroptosis by facilitating *PRNP* translation, thereby contributing to oxaliplatin resistance in colorectal cancer.^[^ ^19^ ^]^ This implies that *PRNP* is a critical gene in ferroptosis resistance and a potential target for creatine. IHC analysis revealed that the ectopic lesions showed higher PrP expression levels than that in the control tissues but no significant difference in ZIP14 expression between groups. Initially, high expression levels of PrP were expected to lead to high iron concentrations and ferroptosis in EESCs. However, in EMs, EESCs prefer to survive and grow rather than succumb to high‐iron environments. Creatine is hypothesized to promote high iron tolerance in HESCs by binding to PrP and interfering with its function, considering that creatine does not influence the expression of *PRNP* or *SLC39A14*.

A change in the conformation of PrP from a mainly α‐helical to a β‐sheet rich PrP‐scrapie (PrP^Sc^) form results in neurotoxicity.^[^ ^27^ ^]^ This suggests that alterations in PrP conformation influence its function. Docking simulations indicated that creatine could potentially bind to the 4 amino acid residues at the active center of PrP via hydrogen bonding. The active site's specificity is pivotal as it selectively engages with particular substrates or reactants, facilitating their transformation. The 3D architecture of this site is fundamental to the enzyme's ability to recognize substrates and its catalytic potency.^[^ ^37^ ^]^ PrP, known to facilitate the reduction of trivalent iron to divalent iron, operates in tandem with ZIP14 as a ferrireductase partner. Creatine binding is suggested to induce conformational shifts within the active site, impairing PrP's catalytic role.

To investigate whether creatine interferes with PrP function, *PRNP*‐overexpressing and si‐*PRNP* HESCs were obtained. Based on these findings, we demonstrated that the overexpression of *PRNP* reduced cell viability by increasing the intracellular free divalent iron and storage iron concentrations, as well as by enhancing the levels of ROS and MDA, thus inducing ferroptosis. However, co‐treatment with creatine mitigated these effects, lowered intracellular iron concentrations, and prevented oxidative stress and lipid peroxidation, thereby enhancing cell survival. Conversely, in si‐*PRNP* HESCs, the concentrations of intracellular free divalent and stored iron decreased, leading to the downregulation of ROS and MDA levels, ultimately promoting cell viability. Meanwhile, creatine supplementation resulted in a loss of capacity to further reduce cellular iron concentrations and protect cells from ferroptosis in si‐*PRNP* HESCs. Hence, we hypothesized that creatine binds to the active center of PrP and inhibits its function, consequently rescuing HESCs from ferroptosis.

Additionally, mice treated with creatine exhibited an increase in the number, weight, and volume of ectopic lesions. In *Prnp*‐overexpressing mice, there was a decrease in the number of ectopic endometrial lesions, whereas supplementation with creatine increased the lesion count. Therefore, we hypothesized that creatine promotes the development of EMs by inhibiting the function of PrP in a mouse model.

Given PrP's membrane localization—either on the cell surface or within endosomes—the current methodological constraints impede an exact evaluation of its catalytic efficiency concerning iron conversion. Further research is essential to map the precise binding site of creatine on PrP, verify its alignment with the predicted active site, and understand the conformational alterations and their implications on PrP functionality. Furthermore, the results were obtained from in vitro cell cultures and EMs mouse models, rendering it challenging to fully simulate the complex in vivo environment and physiological responses. These findings need to be further verified using additional clinical and animal studies.

Based on the findings of this study and those reported in the literature, menstrual tissue fragments flow back into the pelvic and abdominal cavities through retrograde menstruation, and retrograde menstrual blood is rich in heme and iron. Heme overload leads to a decrease in the phagocytic capacity of macrophages and promotes the immune evasion of ectopic foci. However, heme lysis serves as a source of iron overload. Excess trivalent iron is reduced to divalent iron by PrP overexpression and is subsequently absorbed by EESCs, inducing ferroptosis. This dynamic process maintains homeostasis in the pelvic and abdominal microenvironments, thereby preventing the occurrence and progression of EMs. When ESCs express GATM at high levels and accumulate creatine, creatine interacts with the intracellular sensing protein PrP, causing functional abnormalities. This interaction inhibits the conversion of trivalent iron to divalent iron and reduces iron uptake, thereby enhancing the tolerance of ESCs to ferroptosis. Such mechanisms are pivotal in triggering the onset and progression of endometriosis (Figure 7).

This study first presents novel findings on the accumulation of creatine in the peritoneal fluid and EESCs in endometriosis. Although the role of creatine has been extensively investigated in tumors, our research is pioneering in exploring its implications in endometriosis. DARTS assay has enabled us to identify creatine‐sensing proteins and to decipher their contribution to the resistance to ferroptosis in EESCs, which has not been explored in previous endometriosis research. This innovative linkage between creatine and ferroptosis enriches our understanding of endometriosis. Furthermore, our findings suggest that creatine could serve as a promising biomarker for the early diagnosis of endometriosis and highlight novel therapeutic targets for its treatment.

## 4 Experimental Section

### Clinical Samples

This investigation was approved by the Human Research Ethics Committee of the Obstetrics and Gynecology Hospital, Fudan University (No. kyy2024‐157). All participants provided written informed consent. For the control group (without EMs), peritoneal fluid and normal endometrial samples were obtained from patients undergoing dilatation and curettage during the proliferative and secretory phases, with endometrial disease excluded by pathology (*n* = 20; age range, 22−40 years). For the endometriosis group, peritoneal fluid and endometriotic tissue samples were acquired from patients who had undergone laparoscopic surgery for ovarian endometrioma or endometriotic lesions as confirmed by pathology (*n* = 20; age range, 22−40 years).

### Primary Cell Isolation and Cell Culture

Endometrial and endometriotic tissues were sectioned into 1 mm^3^ pieces and digested using 0.5% type IV collagenase (Sigma‐Aldrich, St. Louis, MO, USA; C5138‐1), dissolved in Dulbecco's modified Eagle's medium (DMEM)/F12 (Gibco; Thermo Fisher Scientific, Waltham, MA, USA; 11 330 032), and incubated with constant shaking for 40 min at 37 °C. After digestion, the tissues were filtered through stainless steel sieves ranging from 70 to 40 µm. Centrifuged the cell suspension at 300 × g for 10 min at 4 °C. Discarded the supernatant, then resuspended the cells in complete DMEM/F‐12 medium supplemented with 10% fetal bovine serum (FBS) (Gibco; 10099141C) and 1% penicillin‐streptomycin‐Amphotericin B (NCM Biotech, Suzhou, China; C125C8), and incubated in a humidified atmosphere at 37 °C with 5% CO2 and 20% O2. Purified and cultured NESCs and EESCs from normal endometrium and endometriotic tissues, respectively. HESCs were cultured in complete DMEM/F‐12 medium and maintained in a 37 °C humidified incubator with 5% CO2 and 20% O2.

### Reagents

Creatine (Macklin, Shanghai, China; C804738) was dissolved in PBS (pH 7.4) and sterilized by filtration through a 0.22 µm filter (Millipore, Burlington, MA, USA; SLGP033R). Creatine solution was diluted to different concentrations ranging from 10^2^ to 10^4^ µM in a DMEM/F‐12 medium. Ammonium iron (III) citrate (FAC; Sigma‐Aldrich; F5879) was dissolved and diluted to concentrations ranging from 50 to 400 µM in PBS.

### Metabolomics

Purified NESCs and EESCs were washed thrice with cold PBS, and incubated with 1 mL of 80% (v/v) methanol at −80 °C for 3 h. After centrifugation at 14000 × g for 20 min, the pellet was resuspended in 0.1 M KOH by vortexing and was shaken overnight to dissolve all of the proteins. The metabolite‐containing supernatants were transferred to new 1.5 mL tubes and dried under nitrogen flow. The dried samples were stored at −80 °C for subsequent analysis. Untargeted metabolomics was performed by mass spectrometer (Thermo Fisher Scientific). Data were acquired in the positive and negative ion modes using data‐dependent MS/MS acquisition. The VIP value (threshold > 1) of the Orthogonal Partial Least Squares Discriminant Analysis (OPLS‐DA) model was combined with the *p*‐value from the Student's *t*‐test (*p* < 0.05) to identify differentially expressed metabolites. Differentially expressed metabolites were characterized by searching the Metlin database (https://metlin.scripps.edu; comparing mass‐charge ratios m/z of mass spectra or exact molecular masses).^[^ ^38^ ^]^ The detailed metabolomics raw data was provided in Tables S2 and S3 (Supporting Information).

### qRT‐PCR Analysis

Total RNA was extracted using the EZ‐press RNA purification kit (EZ Bioscience; ZScience Biotechnology Corporation, Roseville, USA; B0004DP) in accordance with the manufacturer's instructions. Subsequently, 1,000 ng RNA was reverse‐transcribed into double‐stranded cDNA using the reverse transcriptional kit (Yeasen Biotech, Shanghai, China; 11141ES60) according to the manufacturer's protocol. Ultimately, qRT‐PCR analysis was conducted using the qPCR SYBR green master mix (Yeasen Biotech; 11184ES08) with an ABI PRISMTM 7900 Sequence Detector (Applied Biosystems; Thermo Fisher Scientific). Table S1 (Supporting Information) presents the detailed sequences of the primers used in this experiment.

### Western Blot Analysis

Cells were first washed with cold PBS and then lysed with radioimmunoprecipitation assay (RIPA) lysis buffer (Beyotime, Shanghai, China; P0013B) supplemented with 1% protease inhibitor cocktail (Yeasen Biotech; 20124ES03). The BCA protein assay kit (Beyotime; P0010) was used to measure the protein concentration. Total protein lysate (20 µg) was loaded on a 10% sodium dodecyl sulfate (SDS)‐gel, separated by sodium dodecyl sulfate‐polyacrylamide gel electrophoresis (SDS‐PAGE), and transferred to PVDF membranes (0.45 µm, EMD Millipore). After blocking with 5% skimmed milk at room temperature for 1 h, the membranes were trimmed according to the markers and the expected band sizes. The membranes were incubated with different antibodies at 4 °C overnight. The antibodies used in this experiment were as follows: Anti‐SLC6A8 (Proteintech, Chicago, USA; 20 299), Anti‐GATM (Abcam, Cambridge, UK; ab238605), Anti‐GAMT (Abcam; ab126736), Anti‐PrP (Abcam; ab52604), Anti‐ZIP14 (Proteintech; 26 540), Anti‐FLAG (Sigma‐Aldrich; F1804), Anti‐Ferritin Heavy Chain (Abcam; ab65080), Anti‐Ferritin Light Chain (Abcam; ab69090). Anti‐α‐tubulin (Proteintech; 11 224) was used as the internal control. The next day, the membranes were washed with TBST (0.1% Tween in Tris‐buffered saline) and incubated with the horseradish peroxidase‐conjugated secondary antibody: goat anti‐rabbit IgG‐HRP antibody (Cell Signaling Technology, Boston, MA, USA; 7074) or horse anti‐mouse IgG‐HRP antibody (Cell Signaling Technology; 7076) for 1 h at room temperature. An enhanced chemiluminescent (ECL) kit (Yeasen Biotech; 36208ES60) was used to visualize the signal. Data analysis was performed with ImageJ software (National Institutes of Health; version 1.8.0).

### Creatine Quantification

Creatine concentration was measured using a creatine assay kit (Abcam; ab65339), following the manufacturer's instructions. After washing cells (2 × 10^6^ cells) with cold PBS, they were resuspended in 100 µL of assay buffer and homogenized by pipetting. Subsequently, cell samples were incubated on ice for 10 to 30 min and then centrifuged for 2–5 min at 4 °C at 13000 × g to remove any insoluble material. The supernatant was collected and transferred to a clean tube. Peritoneal fluid samples were centrifuged for 2–5 min at 4 °C at 13000 × g, and the supernatant was collected and transferred to a clean tube. The cell and peritoneal fluid samples underwent deproteinization by mixing 400 µL of sample with 100 µL ice‐cold Perchloric acid (PCA) and vortexed briefly on ice for 5 min to mix well. After centrifugation at 13000 × g for 2 min, the supernatant was transferred to a fresh tube. Ice‐cold 2 M Potassium Hydroxide (KOH) was used to precipitate the excess PCA and neutralize the samples until the pH reached 6.5–8. The creatine reaction mix was added to both the sample and standard wells. After incubating for 60 min, the creatine concentration was quantified according to the manufacturer's directions using a microplate reader (BioTek Instruments; Thermo Fisher Scientific).

### GEO Data Processing and Identification of Differentially Expressed Genes (DEGs)

The dataset GSE7305 was acquired from the Gene Expression Omnibus (GEO; https://www.ncbi.nlm.nih.gov/geo) database. The GSE7305 dataset comprises data collected from 10 endometriosis and normal uterine endometrium samples, using the GPL570 platform.^[^ ^39^ ^]^ Transcriptome analysis was conducted using R (version 4.2.3) and RStudio software (version RStudio 2023.06.2+561). A probe expression matrix of the raw data was normalized and subsequently converted into a gene expression matrix via the platform annotation file. A merging normalized gene expression matrix was obtained using the limma R package (version 3.56.2) for further analysis. Differential expression analysis was performed for endometriosis and normal samples with the limma R package. DEGs were defined as genes with *p*‐value < 0.5 and |log2 fold change| >1. Volcano plots for all identified genes were generated using the ggplot2 (version 3.4.3), dplyr (version 1.1.3), and ggrepel (version 0.9.3), R packages. A heatmap depicting creatine metabolism‐related genes was created using the heatmap R package (version 1.0.12).

### Measurement of Cell Viability by CCK‐8 Assay

Cell viability was assessed by the cell counting kit‐8 (NCM Biotech; C6005). HESCs were seeded into a 96‐well plate at a density of 4 × 10^3^ cells per well in 100 µL of complete medium at 37 °C for 24 h. The following day, HESCs were treated with 200 µM FAC for 24 h. The supernatant was removed and 100 µL of a complete medium containing 10 µL CCK‐8 solution was added to each well. The plates were incubated at 37 °C for 1 h. A microplate reader (BioTek Instruments; Thermo Fisher Scientific) was used to measure the absorbance at 450 nm.

### ROS Measurement

ROS assay kit (Beyotime; S0033S) was used to detect the level of ROS in normal HESCs using DCFH‐DA probes. ROS assay kit (Solarbio, Beijing, China; CA1420) was used to detect the level of ROS in OE‐*PRNP* HESCs and si‐*PRNP* HESCs using DHE probes. HESCs were seeded into a 6‐well plate containing a complete medium and incubated at 37 °C overnight. After treatment with dimethyl sulfoxide (DMSO), 200 µM FAC, or 200 µM FAC + 1 mM creatine for 24 h, the cells were harvested and washed twice with PBS. The fluorescent probe, either DCFH‐DA or DHE, was loaded at a 1:1000 dilution and incubated in the dark at 37 °C, following the instruction manual. After 30 min, the mean fluorescence intensity (MFI), reflecting the intracellular ROS levels, was measured using a CytoFLEX flow cytometer (Beckman Coulter, Inc., Brea, CA, USA). The data was analyzed with FlowJo (version 10.8.1, BD Life Sciences‐Biosciences, Ashland, OR, USA).

### MDA Measurement

The MDA assay kit (Beyotime; S0131S) was used to detect the concentration of intracellular MDA and to measure the level of intracellular lipid superoxidation. According to the manufacturer's instructions, colorimetry was used to detect MDA in cell lysates based on the color reaction of MDA and thiobarbituric acid (TBA). The MDA levels were measured by Multi‐Mode Microplate Readers (BioTek Instruments; Thermo Fisher Scientific) at 532 nm.

### Wound Healing and Transwell Assays

Cells were seeded in 12‐well plates (2 × 10^5^ cells/well) and cultured until they achieved 80% confluence. A pipette tip was used to create the wound by manually scratching the cells. The cells were cultured in creatine media with concentrations ranging from 0 to 1000 µM for 12 h. An inverted microscope (Olympus Corporation, Tokyo, Japan) was used to capture images at 0 and 12 h. ImageJ was used to measure the wound area at 0 and 12 h. Matrigel (Corning, NY, USA; 356 234) diluted in DMEM (1:8) was added to the upper chamber to coagulate for 12 h at 4 °C. HESCs were seeded into the upper chamber (8 µm pore size, 24‐well plate, Corning) in 200 µL of serum‐free DMEM (2 × 10^5^ cells/well). A complete DMEM medium with 10% FBS was added to the lower chamber as the chemoattractant. Prior to starting the invasion assay, a range of concentrations from 0 to 1000 µM was added to the upper chamber for 24 h. The following day, cells were fixed and stained with 4% paraformaldehyde and crystal violet for 30 min. Finally, the inverted microscope was used to capture images and the number of cells that had invaded were counted.

### DARTS and Mass Spectrometry Analyses

The DARTS assay was performed according to protocols described in previous studies.^[^ ^40^ ^]^ Untreated HESCs were lysed with western blotting or immunoprecipitation lysis buffer supplemented with a 1% protease inhibitor cocktail (Yeasen Biotech; 20124ES03) at 4 °C for 2 h. After centrifugation (12000 × g, 20 min), cell lysates from independent biological replicates were divided into equal volumes, each containing 2,500 µg of protein. They were then incubated at room temperature for 1 h with 100 µM creatine or DMSO. Aliquoted lysates were digested with pronase at a ratio of 1:300 (w/w) for 30 min. Subsequently, a loading buffer was added to the lysates, which were boiled for 10 min. The samples were separated by SDS‐PAGE and stained with Coomassie blue. After the band was excised, destained, reduced, alkylated, and digested with trypsin, the digested product was extracted and desalted using a ZipTip C18 (Millipore; Z720070). After that, vacuum drying and reconstituted were performed. Separation was performed using an EASY‐nLC 1200 system (Thermo Fisher Scientific) using an analytical column (C18, 1.9 µm, 75 µm × 20 cm) at a flow rate of 300 nL min^−1^. Mass spectrometry analysis was conducted using a Q‐Exactive HF mass spectrometer (Thermo Fisher Scientific).

### Immunohistochemical Assay

Normal endometrium and ectopic lesions were fixed in 4% paraformaldehyde, embedded in paraffin, and subsequently sectioned into 4‐µm‐thick sections. Hematoxylin and eosin (H&E) staining of slides was performed to confirm the diagnosis. After antigen retrieval and blocking, sections were incubated overnight with primary antibodies against PrP (Abcam; ab52604) and ZIP14 (Proteintech; 26 540) at 4 °C. The following day, the sections were incubated with HRP‐conjugated goat polyclonal antibody at room temperature for 30 min and developed with diaminobenzidine (DAB). Ultimately, sections were counterstained with hematoxylin for 30 s and sealed with neutral resin. Images were captured using a digital camera‐equipped microscope (Olympus Corporation).

### Molecular Docking

The protein structure of PrP used for docking analysis was downloaded from the AlphaFold Protein Structure Database (https://alphafold.ebi.ac.uk/entry/P04156). The creatine structure was obtained from PubChem Database (https://pubchem.ncbi.nlm.nih.gov). The AutoDock software (https://autodock.scripps.edu) was employed to generate docked conformations of creatine bound to PrP.^[^ ^24^ ^]^ Crystal water and FBP were removed, and hydrogen atoms were added. DoGSiteScorer in Proteins Plus (https://proteins.plus/) was used to predict the active center of PrP.^[^ ^26^ ^]^ The PyMol software (https://pymol.org) was used to visualize the possible structural models of creatine bound to PrP.^[^ ^25^ ^]^

### Lentiviral Transduction

HESCs (5 × 10^5^ cells/well) were seeded in 6‐well plates using complete DMEM and cultured to 70% confluence. Cells were then transfected with *PRNP*‐overexpressing lentiviruses (LV‐*PRNP*) or negative control lentiviruses (GV492 empty vector) (both from Genechem, Shanghai, China) with an optimal multiplicity of infection (MOI) determined to be 10. The supernatant was replaced with a complete culture medium after 12 h. The transfection efficiency can be observed under a fluorescence microscope (Olympus Corporation) after 48 h. The transfection efficiency of *PRNP* was evaluated by qRT‐PCR and western blotting after 72 h. The stably transfected HESCs that overexpress *PRNP* were selected using 10 mg L^−1^ puromycin.

### siRNA Transfection

Negative control, *PRNP*‐724, *PRNP*‐597, *PRNP*‐476, and *PRNP*‐2407 siRNA oligos were specifically designed and procured from GenePharma Company (Shanghai, China). HESCs were transfected with the oligos by Lipofectamine 3000 transfection reagents (Thermo Fisher Scientific; L3000001) following the manufacturer's instructions. After 48 h of transfection, qRT‐PCR and western blotting were used to evaluate the transfection efficiency. siRNA oligo sequences are listed in Table S4 (Supporting Information).

### Mouse Model

Six‐week‐old female C57BL/6 mice were purchased from Chengxi Biotech Company (Shanghai, China). The mice were raised in specific‐pathogen‐free (SPF)–level rooms with free access to food and water. All animal experiments were approved by the Ethics Committee of the Obstetrics and Gynecology Hospital, Fudan University (2024‐FCKYY‐232).

A mouse model of EMs was established by intraperitoneal injection of uterine fragments from the same species, as detailed in previous studies.^[^ ^28^ ^]^ Briefly, mice were designated as donors or recipients (1:1 donor‐to‐recipient ratio). After a week of acclimatization, donor mice received intramuscular injections of 0.3 ug permouse estradiol benzoate once every 3 days to synchronize the estrous cycle. One week later, uterine tissues from the donor mice were minced with scissors to ensure that the maximal diameter of the fragments was consistently smaller than 1 mm^3^. Subsequently, uterine tissue fragments from the donors were randomly injected into the peritoneal cavity of recipient mice. This day was marked as day 0. On day 4, recipient mice were divided into 2 groups: the control group (*n* = 5) received a PBS solution via intraperitoneal injection once every 3 days and the creatine group (*n* = 5) received a creatine solution (13 mg mL^−1^, 100 mM) via intraperitoneal injection once every 3 days. Four weeks later, mice were euthanized by cervical dislocation. The uterus and ectopic tissues were excised for subsequent evaluation. The ectopic lesion tissues were collected, photographed, measured, and weighed. A portion of the collected ectopic lesion tissue was fixed, embedded in paraffin, and stained with Prussian blue.

### AAV‐Prnp Mouse Model

*Prnp*‐overexpression AAVs and NC AAVs were constructed by Genechem (Shanghai, China). The CMV‐betaGlobin‐MCS‐SV40 PolyA was used as a vector. The mice were allocated into 3 groups. The control group (*n *= 8) received an intravenous injection of 200 µL of PBS; the NC group (*n *= 8) received an intravenous injection of the NC AAVs (1 × 10^12^ v.g. mL^−1^, 200 µL); and the OE‐*Prnp* (*Prnp* overexpression) group (*n *= 13) received an intravenous injection of the *Prnp*‐overexpression AAVs (1 × 10^12^ v.g. mL^−1^, 200 µL). Two weeks later, mice received intramuscular injections of 0.3 ug per mouse estradiol benzoate every 3 days. After 1 week, endometrial tissues were harvested. Three endometrial tissues from each group were lysed using RIPA lysis buffer, and western blotting analysis was performed. Another 20 mice were divided into 3 groups (*n *= 5, 5, and 10) and intraperitoneally injected with the remaining uterine tissue fragments from the donor mice (1:1) in the corresponding group. Starting on day 4, the control and NC groups were intraperitoneally injected with PBS solution once every 3 days. The OE‐*Prnp* group was divided into 2 subgroups (*n *= 5 each) on day 4. One group received a PBS solution via intraperitoneal injection once every 3 days (OE‐*Prnp* group), and the other group received a creatine solution (13 mg mL^−1^, 100 mM) by intraperitoneal injection once every 3 days (OE‐*Prnp* + Cr group). Two weeks later, the mice were euthanized by cervical dislocation. The ectopic lesion tissues were harvested, photographed, measured, and weighed.

### Statistical Analysis

All experiments were conducted to include 3 samples per condition, and each experiment was independently repeated more than 3 times. All data were expressed as the mean ± standard error of the mean (SEM), with n indicating the number of experiments or animals analyzed. Statistical analyses were performed using GraphPad Prism Software (GraphPad Software Inc). Types of statistical tests were indicated in figure legends. The data were first tested for normality. Two‐tailed Student's *t*‐test was used in a two‐group comparison, and Welch's correction was used when the F‐test showed a significant difference. One‐way analysis of variance (ANOVA) was used to compare data from more than 2 groups under a single treatment, whereas two‐way ANOVA was employed to compare data from more than 2 groups under multiple treatments. If the data did not follow a normal distribution, the Mann‐Whitney test was used in the two‐group comparison. Values of *p *< 0.05 were considered statistically significant.

## Conflict of Interest

The authors declare no conflict of interest.

## Author Contributions

S.C. and X.M. contributed equally to this work. S.C. and X.M. conducted all the experiments and arranged the figures and the manuscript. Y.L. assisted with sample collection. Z.Z. provided technological support for biochemical experiments. C.W. assisted with the animal experiments. M.L. and X.Z. initiated and supervised the project and edited the manuscript. All authors approved the final manuscript.

## Supporting information

Supporting Information

## Data Availability

The data that support the findings of this study are available from the corresponding author upon reasonable request.
