# Supplementary material for: Creatine Promotes Endometriosis by Inducing Ferroptosis Resistance via Suppression of PrP
Source: Adv Sci (Weinh). 2024 Aug 9;11(38):2403517. doi: 10.1002/advs.202403517 (PMC11481182; doi:10.1002/advs.202403517)
Supplement: Supplementary file 1 — Supporting Information [file ADVS-11-2403517-s001.docx]

Supporting Information

Creatine Promotes Endometriosis by Inducing Ferroptosis Resistance via Suppression of PrP

Siman Chen, Xiaoqian Ma, Yukai Liu, Zhiqi Zhong, Chunyan Wei, Mingqing Li,* and Xiaoyong Zhu*

**Table S1.** List of all primer sequences used for quantitative reverse transcription-polymerase chain reaction (qRT-PCR).

| Gene name | Forward and reverse primer | Tm [◦C] |
| --- | --- | --- |
| *PRNP* | F: 5’ AGTCAGTGGAACAAGCCGAG 3’  R: 5’ CTGCCGAAATGTATGATGGGC 3’ | 61.8  61.4 |
| *SLC39A14* | F: 5’ AAGGCCCTACTCAACCACCT 3’  R: 5’ CGACTGCTCGCTGAAATTGTG 3’ | 62.4  62.1 |
| *SLC6A8* | F: 5’ GGCCTGGGGCTTCTATTACC 3’  R: 5’ CAGCCTCAAGACTTTGTTCTCC 3’ | 61.8  60.8 |
| *GATM* | F: 5’ CACTACATCGGATCTCGGCTT 3’  R: 5’ CTAAGGGGTCCCATTCGTTGT 3’ | 61.4  61.5 |
| *GAMT* | F: 5’ CGCCCATTGATGAGCATTGG 3’  R: 5’ GGCCTTTCAAGGGGATGACC 3’ | 61.8  62.6 |
| *FTH1* | F: 5’ CCCCCATTTGTGTGACTTCAT 3’  R: 5’ GCCCGAGGCTTAGCTTTCATT 3’ | 60.2  62.8 |
| *FTL* | F: 5’ CAGCCTGGTCAATTTGTACCT 3’  R: 5’ GCCAATTCGCGGAAGAAGTG 3’ | 60.0  62.0 |
| *GAPDH* | F: 5’ GGAGCGAGATCCCTCCAAAAT 3’  R: 5’ GGCTGTTGTCATACTTCTCATGG 3’ | 61.6  60.9 |

**Table S2.** The list of differential metabolites of normal endometrial stromal cells (NESCs) and ectopic endometrial stromal cells (EESCs) at positive ion mode.

| name | VIP | CompMW | Time | p value | fold change |
| --- | --- | --- | --- | --- | --- |
| Acetylcarnitine | 1.757 | 203.1159 | 0.95 | 0.004 | -1.470 |
| Creatine | 1.981 | 131.0695 | 0.90 | 0.001 | -1.829 |
| Glycerophosphocholine | 1.610 | 257.1026 | 1.10 | 0.011 | 1.608 |
| Kynurenine | 2.023 | 208.0849 | 1.95 | 0.001 | 2.282 |
| L-Carnitine | 1.792 | 161.1052 | 0.90 | 0.003 | -1.230 |
| L-Glutamate | 1.888 | 147.0531 | 0.90 | 0.002 | -1.425 |
| L-Lysine | 1.636 | 146.1055 | 0.80 | 0.009 | 0.263 |
| L-Proline | 2.024 | 115.0633 | 0.99 | 0.001 | -0.673 |
| LysoPC(16:1(9Z)) | 1.348 | 493.3169 | 7.22 | 0.037 | -0.602 |
| PA(18:3(9Z,12Z,15Z)/20:5(5Z,8Z,11Z,14Z,17Z)) | 2.139 | 716.3761 | 3.52 | 0.000 | -1.711 |
| Pantothenic Acid | 1.439 | 219.1108 | 2.41 | 0.025 | -0.495 |
| PIP(20:0/18:2(9Z,12Z)) | 1.397 | 970.5550 | 1.21 | 0.030 | 1.088 |
| Acetylcholine | 2.083 | 145.1101 | 0.93 | 0.000 | -1.131 |
| cAMP | 1.591 | 329.0530 | 1.21 | 0.012 | -4.317 |
| L-Threonine | 1.371 | 119.0580 | 0.90 | 0.034 | -0.317 |
| LysoPC(14:0) | 1.495 | 467.3008 | 6.90 | 0.019 | -0.812 |
| LysoPC(15:0) | 1.965 | 481.3168 | 7.45 | 0.001 | -1.822 |
| LysoPC(18:1(11Z)) | 1.545 | 521.3488 | 8.31 | 0.015 | -0.898 |
| LysoPC(18:2(9Z,12Z)) | 1.354 | 519.3332 | 7.55 | 0.036 | -0.666 |
| Adrenosterone | 1.356 | 300.1717 | 3.51 | 0.036 | 1.600 |
| Choline | 1.281 | 103.0995 | 0.87 | 0.049 | -0.204 |
| DG(20:3(5Z,8Z,11Z)/20:4(5Z,8Z,11Z,14Z)/0:0) | 1.489 | 666.5206 | 13.25 | 0.020 | 1.487 |
| β-Alanine | 1.352 | 89.0474 | 0.89 | 0.037 | -0.468 |
| PI(18:4(6Z,9Z,12Z,15Z)/16:0) | 1.599 | 830.4978 | 3.98 | 0.011 | -2.524 |
| PI(18:4(6Z,9Z,12Z,15Z)/19:1(9Z)) | 2.404 | 870.5295 | 3.75 | 0.000 | 2.681 |

VIP, variable importance in the projection; CompMW, component molecular weight; foldchange, log2foldchange (NESN/EESC), a positive sign indicating a rise in NESCs group relative to EESCs group and a negative sign indicating a reduction.

**Table S3.** The list of differential metabolites of NESCs and EESCs at negative ion mode.

| name | VIP | CompMW | Time | p value | fold change |
| --- | --- | --- | --- | --- | --- |
| Citric acid | 2.035 | 192.0271 | 1.21 | 0.000 | -0.910 |
| Dehydroascorbic acid | 1.994 | 174.0166 | 1.21 | 0.000 | -1.415 |
| Gluconic acid | 1.228 | 196.0584 | 0.89 | 0.046 | -0.583 |
| Kynurenine | 1.856 | 208.0846 | 2.07 | 0.001 | 3.143 |
| L-Glutamate | 1.880 | 147.0535 | 0.90 | 0.001 | -1.628 |
| Linoleic acid | 1.280 | 280.2400 | 10.11 | 0.036 | 1.119 |
| L-Tyrosine | 1.578 | 181.0739 | 1.26 | 0.007 | -0.439 |
| Oleic Acid | 1.251 | 282.2558 | 8.05 | 0.041 | -0.828 |
| Oxoglutaric acid | 2.035 | 146.0216 | 1.20 | 0.000 | -0.910 |
| PG(18:2(9Z,12Z)/0:0) | 1.309 | 508.2797 | 8.00 | 0.032 | 1.233 |
| PG(20:4(5Z,8Z,11Z,14Z)/0:0) | 1.723 | 532.2799 | 8.00 | 0.003 | 2.843 |
| Uridine diphosphate (UDP) | 1.793 | 404.0019 | 1.10 | 0.002 | -4.453 |
| 9(S)-HOTrE | 1.433 | 294.2192 | 8.19 | 0.017 | 3.216 |
| Citramalic acid | 1.887 | 148.0374 | 1.27 | 0.001 | -1.810 |
| D-Sorbitol | 1.266 | 182.0792 | 0.85 | 0.039 | -2.503 |
| Fumaric acid | 1.232 | 116.0113 | 0.93 | 0.045 | -0.541 |
| Guanosine 5′-diphosphate (GDP) | 1.773 | 443.0238 | 1.14 | 0.002 | -2.918 |
| L-Threonine | 1.611 | 119.0585 | 0.86 | 0.006 | -0.388 |
| LysoPE(0:0/16:0) | 1.552 | 453.2850 | 6.92 | 0.009 | -0.741 |
| LysoPE(0:0/18:1(9Z)) | 1.561 | 479.3005 | 7.24 | 0.008 | -0.667 |
| LysoPE(0:0/18:2(9Z,12Z)) | 1.454 | 477.2850 | 6.90 | 0.015 | -0.871 |
| LysoPE(0:0/22:5(4Z,7Z,10Z,13Z,16Z)) | 1.250 | 527.3006 | 7.61 | 0.041 | -1.028 |
| LysoPE(0:0/24:6(6Z,9Z,12Z,15Z,18Z,21Z)) | 1.372 | 553.3159 | 7.46 | 0.023 | -1.328 |
| LysoPE(20:1(11Z)/0:0) | 1.545 | 507.3318 | 8.33 | 0.009 | -1.034 |
| LysoPE(20:2(11Z,14Z)/0:0) | 1.523 | 505.3160 | 7.58 | 0.010 | -0.973 |
| Malic acid | 1.961 | 134.0217 | 0.93 | 0.000 | -2.964 |
| N-Acetyl-L-glutamic acid | 1.919 | 189.0639 | 0.99 | 0.001 | -2.937 |
| PE(P-18:0/0:0) | 1.281 | 465.3212 | 9.31 | 0.036 | -0.573 |
| PI(16:0/0:0) | 1.540 | 572.2955 | 7.72 | 0.009 | -1.190 |
| PI(18:0/0:0) | 1.359 | 600.3263 | 9.61 | 0.025 | -0.974 |
| PI(18:1(9Z)/0:0) | 1.985 | 598.3109 | 8.00 | 0.000 | -1.562 |
| PI(22:2(13Z,16Z)/0:0) | 1.915 | 652.3528 | 3.30 | 0.001 | -1.512 |
| PS(16:0/0:0) | 1.361 | 497.2745 | 7.83 | 0.025 | 0.822 |
| PS(18:0/0:0) | 1.316 | 525.3059 | 9.08 | 0.031 | 1.145 |
| PS(19:0/0:0) | 1.427 | 539.3214 | 7.26 | 0.018 | -0.689 |
| PS(21:0/0:0) | 1.534 | 567.3525 | 8.33 | 0.010 | -1.028 |
| UDP-glucose | 1.690 | 566.0540 | 0.81 | 0.004 | -1.781 |
| UDP-N-acetyl-D-galactosamine | 1.535 | 607.0805 | 0.81 | 0.010 | -1.403 |
| D-Urobilin | 1.446 | 588.2931 | 4.03 | 0.016 | -1.826 |
| PI(20:4(5Z,8Z,11Z,14Z)/0:0) | 1.333 | 620.2949 | 7.33 | 0.028 | -0.626 |
| PS(18:4(6Z,9Z,12Z,15Z)/22:6(4Z,7Z,10Z,13Z,16Z,19Z)) | 1.467 | 827.4718 | 3.98 | 0.014 | 6.861 |
| Pyroglutamic acid | 1.908 | 129.0428 | 1.21 | 0.001 | -2.279 |
| Glyceric acid | 1.880 | 106.0269 | 0.91 | 0.001 | -1.628 |
| NADH | 1.500 | 665.1241 | 1.21 | 0.012 | -1.694 |
| PC(19:3(10Z,13Z,16Z)/0:0) | 1.490 | 531.3309 | 7.96 | 0.013 | -1.240 |
| PE(P-16:0/22:5(7Z,10Z,13Z,16Z,19Z)) | 1.476 | 749.5389 | 11.13 | 0.014 | 1.157 |
| PI(18:4(6Z,9Z,12Z,15Z)/16:0) | 1.289 | 830.4985 | 3.99 | 0.035 | -3.222 |
| PI(22:0/0:0) | 2.008 | 656.3926 | 3.47 | 0.000 | 2.355 |
| Taurocholic acid | 1.237 | 515.2952 | 3.80 | 0.044 | 1.056 |

**Table S4.** The list of small interfering RNA (siRNA) oligos.

| Sites | Sense (5’--3’) | Antisense (3’--5’) |
| --- | --- | --- |
| Negative control | UUCUCCGAACGUGUCACGUTT | ACGUGACACGUUCGGAGAATT |
| *PRNP*-724 | GGGAAUCUCAGGCCUAUUATT | UAAUAGGCCUGAGAUUCCCTT |
| *PRNP*-597 | GACUGCGUCAAUAUCACAATT | UUGUGAUAUUGACGCAGUCTT |
| *PRNP*-476 | CAUCAUACAUUUCGGCAGUTT | ACUGCCGAAAUGUAUGAUGTT |
| *PRNP*-2407 | GACGAACAGAUUUCAACAATT | UUGUUGAAAUCUGUUCGUCTT |

**Table S5.** The list of 65 potential target proteins of creatine.

| Number | Name | Unique Peptides | Fold change | P value |
| --- | --- | --- | --- | --- |
| 1 | CSTA | 1 | 26.283 | 0 |
| 2 | ACAP2 | 1 | 19.147 | 0.00486 |
| 3 | GID8 | 2 | 4.0403 | 0.03026 |
| 4 | MARCKS | 1 | 3.7229 | 0.00398 |
| 5 | DDX54 | 2 | 3.2789 | 0.00129 |
| 6 | S100A8 | 3 | 3.2304 | 0.02299 |
| 7 | DCTN2 | 3 | 2.5829 | 0.0431 |
| 8 | RTL6 | 1 | 2.564 | 0.03205 |
| 9 | MT1X | 3 | 2.5604 | 0.00382 |
| 10 | NDUFAF2 | 1 | 1.82 | 0.02656 |
| 11 | FAT4 | 3 | 1.7925 | 0.0332 |
| 12 | PRNP | 2 | 1.7694 | 0.00135 |
| 13 | DCTPP1 | 3 | 1.6552 | 0.04637 |
| 14 | CCSER1 | 1 | 1.6113 | 0.04121 |
| 15 | APOA1 | 1 | 1.606 | 0.00066 |
| 16 | AHSA1 | 3 | 1.5754 | 0.00241 |
| 17 | MANEA | 1 | 1.559 | 0.03604 |
| 18 | RGS10 | 2 | 1.5498 | 0.03163 |
| 19 | SMAP2 | 1 | 1.5251 | 0.04241 |
| 20 | BCAS2 | 1 | 1.5209 | 0.04589 |
| 21 | PARG | 1 | 1.4812 | 0.04604 |
| 22 | RAB5C | 4 | 1.4604 | 0.01119 |
| 23 | AHSG | 3 | 1.4436 | 0.0179 |
| 24 | RALGAPA2 | 1 | 1.4431 | 0.02741 |
| 25 | ERH | 4 | 1.4372 | 0.00968 |
| 26 | CCAR2 | 7 | 1.4369 | 0.01512 |
| 27 | ALDOC | 2 | 1.4135 | 0.04875 |
| 28 | TPM3 | 3 | 1.4046 | 0.01089 |
| 29 | ATXN2L | 4 | 1.402 | 0.03011 |
| 30 | H1-3 | 1 | 1.3815 | 0.00299 |
| 31 | CD44 | 7 | 1.3552 | 0.04416 |
| 32 | STAM | 2 | 1.3446 | 0.01285 |
| 33 | CNN2 | 5 | 1.3332 | 0.00336 |
| 34 | PDCD1LG2 | 2 | 1.3315 | 0.03637 |
| 35 | APP | 7 | 1.3303 | 0.02379 |
| 36 | YBX1 | 3 | 1.3274 | 0.01882 |
| 37 | PODXL | 4 | 1.3267 | 0.01581 |
| 38 | FAM234A | 1 | 1.3148 | 0.01916 |
| 39 | SLC39A14 | 2 | 1.305 | 0.00545 |
| 40 | GGT7 | 3 | 1.2926 | 0.04664 |
| 41 | ARFIP2 | 1 | 1.2826 | 0.02066 |
| 42 | CD82 | 2 | 1.281 | 0.00894 |
| 43 | UBQLN1 | 1 | 1.2788 | 0.03201 |
| 44 | TSPAN4 | 4 | 1.2778 | 0.02895 |
| 45 | PUS7L | 1 | 1.2759 | 0.0401 |
| 46 | EEF1B2 | 2 | 1.274 | 0.00914 |
| 47 | VASN | 5 | 1.2651 | 0.04575 |
| 48 | OSTF1 | 2 | 1.2625 | 0.04317 |
| 49 | FEN1 | 4 | 1.2584 | 0.03786 |
| 50 | NPC2 | 6 | 1.2562 | 0.0143 |
| 51 | CD55 | 6 | 1.2516 | 0.04645 |
| 52 | COMMD9 | 2 | 1.2487 | 0.0088 |
| 53 | RTL8A | 1 | 1.2386 | 0.00853 |
| 54 | SUMO2 | 2 | 1.2371 | 0.0354 |
| 55 | P2RX4 | 2 | 1.234 | 0.01744 |
| 56 | S100A10 | 2 | 1.2311 | 0.0257 |
| 57 | SMARCA5 | 4 | 1.2235 | 0.01272 |
| 58 | SGTA | 6 | 1.2169 | 0.04514 |
| 59 | LAMC1 | 28 | 1.2136 | 0.00262 |
| 60 | CNN3 | 2 | 1.2135 | 0.03828 |
| 61 | PIP5K1C | 1 | 1.211 | 0.04051 |
| 62 | RPL36 | 3 | 1.2056 | 0.01309 |
| 63 | TIMM50 | 3 | 1.2014 | 0.01713 |
| 64 | S100A6 | 3 | 1.2003 | 0.01399 |
| 65 | RTF1 | 1 | 1.2001 | 0.0038 |


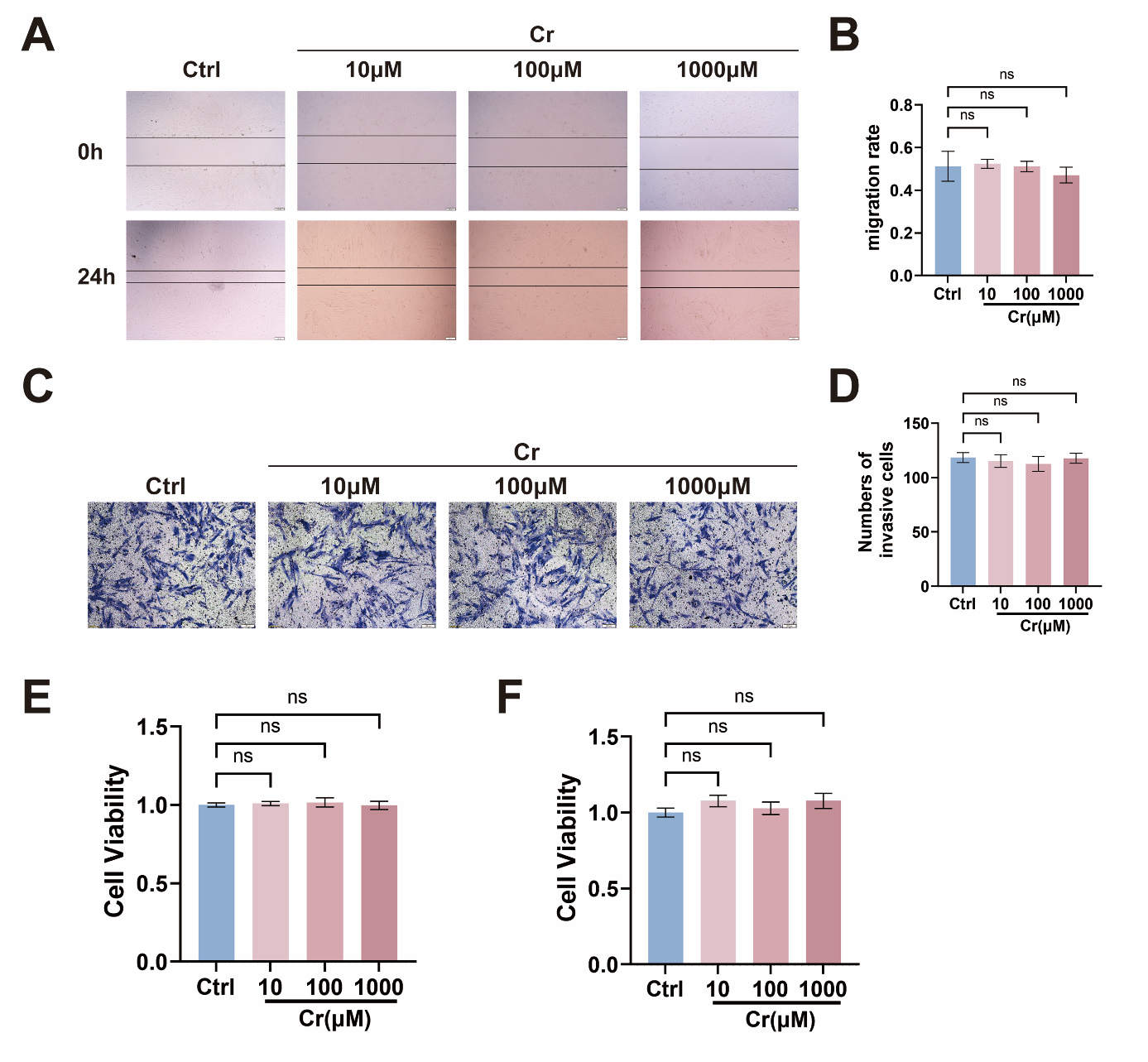


**Figure S1.** Creatine shows no effect on the migration, invasion, and viability of human endometrial stromal cells (HESCs).

A) The wound healing assay was used to assess the effect of a 24-hour treatment with phosphate-buffered saline (PBS) and a range of creatine concentrations on the migratory ability of HESCs. Scale bar, 200 μm. Magnification, 40 ×.

B) Quantitative assessment of migration rate.

C) Transwell assay shows the invasion of HESCs after treatment with PBS and different concentrations of creatine for 24 h. Scale bar, 100 μm. Magnification, 100 ×.

D) Quantitative assessment of invasive cell numbers.

E,F) After treatment with 0, 10, 100, and 1,000 μM creatine media for 24 h or 48 h respectively, CCK-8 assays were used to explore the effect of creatine on HESCs cell viability.

Data in (B,D−F) are presented as the mean ± standard error of mean (SEM), ns, no significant difference by one-way analysis of variance (ANOVA) test.


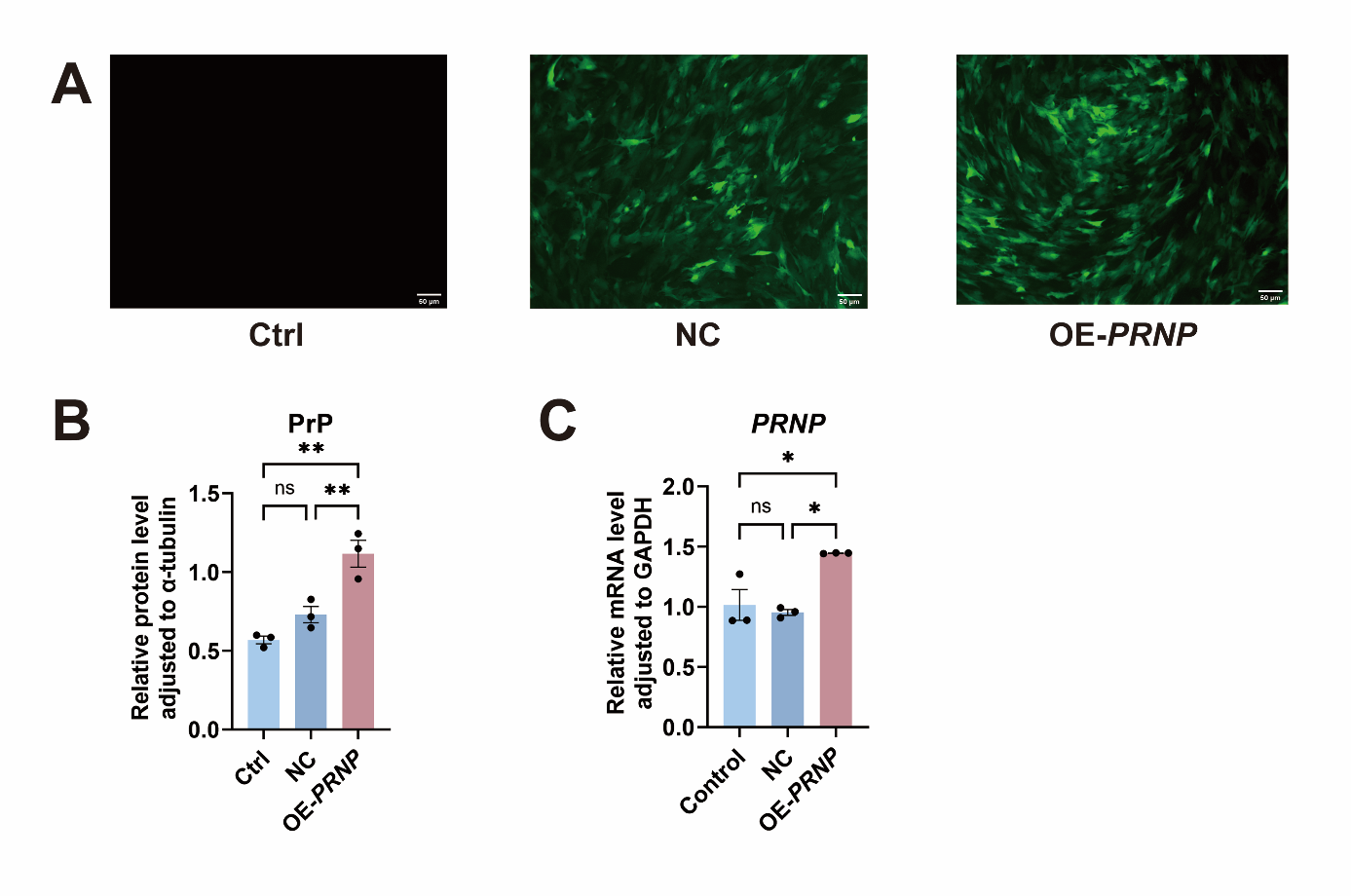


**Figure S2.** The expression levels of prion protein (PrP, gene name *PRNP*) were increased in OE-*PRNP* HESCs.

A) Fluorescent images of green fluorescent protein (GFP) in HESCs after transfection with *PRNP*-overexpressing lentiviruses for 48 h.

B) Densitometry analysis of protein expression levels in Figure 4D quantified using ImageJ.

C) Evaluation of *PRNP* expression levels in HESCs by qRT-PCR.

Data in (B,C) are presented as the mean ± SEM, **p* < 0.05, ***p* < 0.01, ns, no significant difference by one-way ANOVA test.
